# Identification of duck type II interferon-stimulated genes and revelation of *duIFI35* inhibition of H5N6 AIV replication by promoting apoptosis

**DOI:** 10.1186/s13567-026-01747-5

**Published:** 2026-04-16

**Authors:** Tao Zhang, Na Yang, Lulu Ma, Fengxiang Xu, Xiaobing Lin, Jiangwu Huang, Fei Gao, Ming Liao, Min Feng, Manman Dai

**Affiliations:** 1 Guangdong Laboratory for Lingnan Modern Agriculture, National and Regional Joint Engineering Laboratory for Medicament of Zoonosis Prevention and Control, National Avian Influenza Para-Reference Laboratory, College of Veterinary Medicine, South China Agricultural University, Guangzhou, 510642 China; 2 Guangdong Provincial Key Laboratory of Agro-Animal Genomics and Molecular Breeding, College of Animal Science, South China Agricultural University, Guangzhou, 510642 China; 3 UK-China Centre of Excellence for Research on Avian Diseases, Guangzhou, 510642 China

**Keywords:** Duck interferon-stimulated gene, IFN-γ, H5N6 Avian influenza virus, IFI35, apoptosis

## Abstract

H5N6 highly pathogenic avian influenza virus (HPAIV) poses a serious threat to both poultry and public health due to its ability to cross species barriers. Although interferon-stimulated genes (ISGs) are key components of the host’s antiviral defense, a systematic identification and functional characterization of duck ISGs has not yet been conducted. In this study, we identified 815 potential duck ISGs induced by type II interferon (IFN-γ) in duck embryo fibroblasts (DEFs). The majority of these type II ISGs were enriched in immune-related pathways, including “cytokine–cytokine receptor interaction” and “influenza A”. Functional validation using siRNA-mediated knockdown demonstrated that six ISGs, including *duIFI35*, promote H5N6 AIV replication when silenced. Through TUNEL assay, flow cytometry, and apoptotic pathway analysis, Our analysis revealed that H5N6 AIV infection markedly upregulates apoptotic genes such as *Fas*, *FADD*, *caspase-8*, *BAK*, *cytochrome c*, *APAF1*, *caspase-9*, and *caspase-3* (*P* < 0.05), thereby promoting apoptosis in DEFs. In investigating the antiviral mechanism of *duIFI35*, it was found that overexpression of *duIFI35* further enhanced H5N6-induced apoptosis, as evidenced by increased transcription of these apoptotic genes, whereas *duIFI35* knockdown had the opposite effect. Importantly, the antiviral effect of *duIFI35* was significantly diminished upon treatment with the caspase inhibitor Z-VAD-FMK (20 μM) (*P* < 0.05), indicating that its antiviral activity is mediated through apoptosis induction. Collectively, this study provides the first systematic identification of type II ISGs in ducks and reveals *duIFI35*-mediated apoptosis as a critical antiviral mechanism, offering foundational insights into ISG-driven innate immunity against AIV in waterfowl.

**Graphical Abstract:**

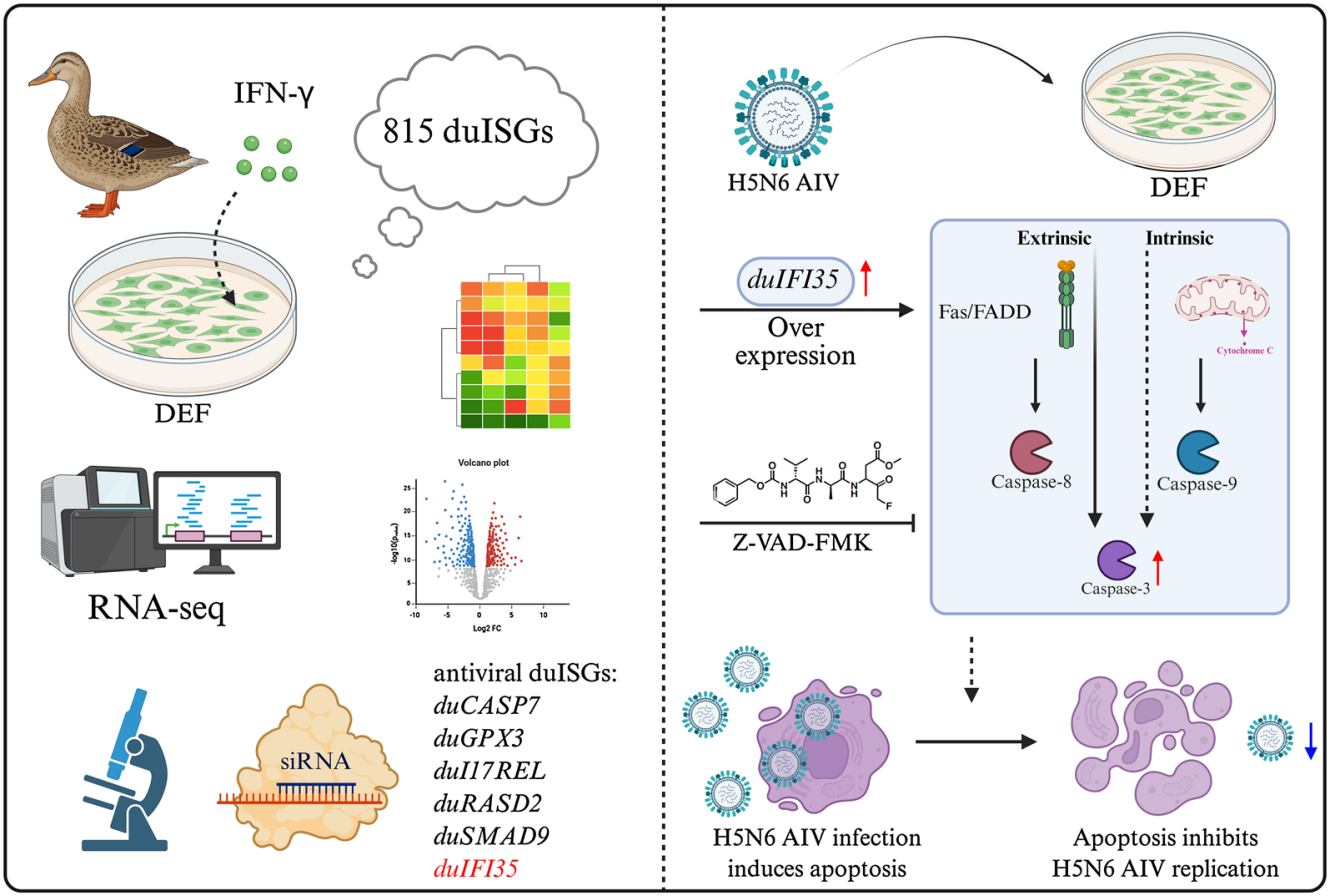

**Supplementary Information:**

The online version contains supplementary material available at 10.1186/s13567-026-01747-5.

## Introduction

The H5N6 avian influenza virus (AIV) was initially isolated from mallard ducks in 1975 and was found to be low in pathogenicity at the time of its discovery. However, by 2021, the recombinant H5N6 AIV had begun to infect humans, demonstrating high pathogenicity with a mortality rate as high as 33% [1]. Since 2020, H5Nx viruses have caused unprecedented global outbreaks, affecting 67 countries and resulting in the loss of over 131 million birds through mortality or culling by 2022 [2]. A study has revealed that ducks, serving as natural reservoirs for influenza A viruses, are capable of replicating most viral strains while mounting robust innate immune responses against highly pathogenic viruses [3]. This tolerance is attributed to its robust innate immune defense, particularly the interferon (IFN)-mediated response. The innate immune system constitutes the first line of defense against viral invasion. It has been reported that avian influenza-triggered innate immunity involves cytokines such as IFNs, and interleukins [4], yet H5N6 AIVs can evade these defenses through mechanisms like transcriptional readthrough [5]. Therefore, understanding how duck-specific immune factors counteract viral escape strategies is crucial for elucidating host–pathogen interactions and developing targeted interventions.

IFNs, a class of cytokines central to antiviral defense, are categorized into three types (I, II, and III) based on receptor specificity and sequence homology [6]. Type I IFNs consist of over 20 members, whereas type II IFNs have only one member, IFN-γ. As a broad-spectrum antiviral cytokine, IFN works not directly against pathogens but by binding to specific receptors on the surface of uninfected cells, which in turn stimulates the production of hundreds of interferon-stimulated genes (ISGs) [7]. In avian species, IFN-γ-mediated ISGs (type II ISGs) play pivotal roles in controlling AIV infections. It has been shown that the antiviral response to H5N6 in chickens can be modulated by targeting IFN-γ through ISGs [8]. Given the antiviral properties of type II ISGs, they have been extensively investigated. A study has demonstrated that IFN-γ-mediated ISGs exert pivotal antiviral effects in Newcastle disease virus (NDV)-infected chicken fibroblasts [9]. Moreover, existing studies have demonstrated that recombinant duck IFN-γ inhibits AIV replication both in vitro and in vivo [10]. Numerous studies have shown that influenza infection is closely related to the production of IFN, the release of pro-inflammatory cytokines, the expression of ISGs, the recruitment of innate immune cells, and the activation of programmed cell death such as apoptosis [11–13]. However, as ducks are natural hosts of H5N6 AIV, research on the identification of duck type II ISGs and their antiviral mechanisms remains insufficient, warranting further investigation.

Given that the specific antiviral mechanisms of type II ISGs in ducks remain unclear, this study systematically identified and functionally validated these genes in duck embryo fibroblasts (DEFs) to unravel the complexities of waterfowl innate immunity.

## Materials and methods

### Methods for primary cell culture

DEFs were prepared from 9 to 11-day-old specific pathogen-free (SPF) duck embryos (National Poultry Laboratory Animal Resource Center). In brief, eggshells were disinfected and opened, and then washed three times with phosphate-buffered solution (PBS). After removing the head, extremities, and viscera, the remaining muscle tissues were cut with sterile scissors and then washed three times with PBS. Clipped tissues were digested in 0.25% trypsin for approximately 10 min at 37 °C in a water bath. After digestion, filtered through a 70 μm filter and centrifuged. The precipitate was re-suspended in Dulbecco’s modified Eagle’s medium (DMEM; Gibco, USA, 12491015) supplemented with 10% fetal bovine serum (FBS; TransGen, China, FS401), 100 U/mL penicillin and 100 mg/mL streptomycin (Gibco, USA, 15140148) at 37 °C in a 5% CO_2_ incubator.

### Virus

All experiments were carried out in an animal biosafety level 3 laboratory and animal facility in compliance with an approved protocol (CNAS BL0011) by the biosafety committee of South China Agriculture University (Guangzhou, China). The H5N6 subtype avian influenza virus, A/Duck/Ji an/J19/2022 (H5N6 subtype AIV) was isolated and stored in the laboratory. The virus was propagated in the allantoic cavities of 9–11-day-old SPF embryonated chicken eggs. The 50% tissue culture infectious dose (TCID_50_) of A/Duck/Ji an/J19/2022 was calculated in Madin-Darby Canine Kidney (MDCK) cells. Assay results were calculated according to the method of Reed & Muench.

The recombinant fluorescent vesicular stomatitis (VSV-GFP) virus was propagated in DEFs [14]. When the cytopathic effect (CPE) reached 90%, the freeze-thawed cell lysate was filtered and collected for later use, and viral titers were determined by TCID_50_ testing.

### IFN-γ antiviral activity assay

Recombinant duck IFN-γ (rDuIFN-γ) was synthesized by Sino Biological (Beijing, China). The activity of rDuIFN-γ protein was analyzed by inhibiting the VSV-induced CPE on DEFs. In brief, DEFs were seeded in 96-well plates and were treated with purified rDuIFNγ diluted fourfold with DMEM medium containing 2% FBS. After 12 h, the supernatant was removed, and the cells were washed with PBS. Attached cells were then infected with 100 TCID_50_ VSV-GFP for 48 to 72 h. The CPE was observed under a microscope, and the number of CPE wells was statistically analyzed. Cell wells containing rDuIFN-γ without VSV were used as negative controls. Cells treated by VSV but lacking rDuIFN-γ were used as positive controls. The reciprocal of the highest dilution of IFN-γ that inhibited 50% CPE was determined to be the unit of anti-VSV-GFP activity. Assay results were calculated according to the method of Reed & Muench and expressed as UI/mg. In addition, IFN-γ activity against H5N6 AIV was also tested by the same method.

### Library preparation for RNA-Seq

After growing into a single layer in a 12-well cell culture plate, DEFs were treated with IFN-γ (1000 UI/mL). At the same time, control groups were set up in which DEFs were treated with DMEM containing 2% FBS. After incubating for 6 h and 24 h, cells were harvested respectively for mRNA transcriptome sequencing. Samples were collected from three independent experiments. RNA-seq was entrusted to Gene Denovo Biotechnology Co. (Guangzhou, China), and the mRNA high-throughput sequencing was completed using the Illumina Hiseq platform.

### Analysis of RNA-Seq data

To ensure high-quality sequencing output, raw reads were filtered to remove sequences containing adapters and low-quality bases, including those with more than 10% unknown nucleotides and those with over 50% low-quality bases (Q-value ≤ 20). High-quality reads were then aligned to the duck reference genome using HISAT2 (version 2.2.1). The mapped read sequences for each sample were reconstructed and assembled using StringTie software [15]. Gene abundance was quantified with RSEM software [15], and gene expression levels were normalized using FPKM (Fragments Per Kilobase of transcript per Million mapped reads). Genes with a fold change (FC) satisfying |log_2_FC|> 1 and a false discovery rate (FDR) < 0.05 were considered differentially expressed genes (DEGs) between groups (IFN-γ vs NC).

All DEGs were mapped to the Gene Ontology (GO) terms in the GO database, and the number of genes in each entry was calculated. GO terms significantly enriched in DEGs, compared to the genomic background, were identified using hypergeometric tests. For pathway enrichment analysis, DEGs were mapped to the Kyoto Encyclopedia of Genes and Genomes (KEGG) database. In this study, upregulated DEGs were identified as duck ISGs.

### Overexpression and knockdown of target genes via transfection

The *duIFI35* gene, amplified by PCR from DEFs, was cloned into the pCAGGS vector to construct the pduIFI35-HA plasmid fragment. The siRNA sequences targeting *CASP7*, *GPX3*, *SAMD9*, *IL17REL*, *RSAD2*, and *IFI35* were designed and synthesized, with the synthesis being commissioned to GenePharma Company.

Cell transfection was performed using PlusTransTM Transaction Reagent (NULEN biotechnology, China, CT801) according to the manufacturer’s instructions. In brief, an appropriate dose of transfection reagents and plasmids or siRNA was separately diluted into 100 µL of OPTI-MEM and incubated at room temperature (25 ℃) for 5 min. Subsequently, the diluents were mixed together and incubated at room temperature for 20 min. The mixture was then added into DEFs.

### Quantitative real-time PCR

Cells were lysed and total RNAs were extracted using RNA Extraction Kit (Vazyme, China) according to the manufacturer’s instructions. Then cDNA was synthesized via reverse transcription using reverse transcriptase (Accurate Biology, China, CM0267) according to the manufacturer’s protocol.

The specific qPCR primers were designed using the National Center for Biotechnology Information (NCBI) Primer BLAST program. As an internal control the duck β-actin gene was used. qPCR results are representative of three independent experiments. Data analyses were performed using the 2^–ΔΔCt^ method. The primer sequences are listed in Table 1.

**Table 1:** **Primer sequences for target genes**

Gene	5′-Primer (F)	3′-Primer (R)
IFI35	GCTCAGCTCTCATCACCTT	GACCGGACACTAGGATGCTC
TRIM25	CTTTCCCCTACGCTGGTCTC	TTGGTGCTCGGTTATCTGCC
C1S	GAAGGTACTATCCTGTCTAGGTG	AGTAGGCTGCAAAACCAGTGA
C1R	AACTTGGGCCGATACTGTGG	GCAAATGTGCTGACACCGAG
LOC101795565	AATCAAGCCTGTCCTCCCCA	ATCGCTGCCACATGAAGACT
ERAP1	ACCACATATCGGGAATCCGC	CCACTGATGAGCCAGTTCGT
NUB1	GCAGCTGGACATAGTGTGGT	GTGGTTCTCCCCATAGCACC
MYOIC	TAAATGACCCTTTCGCGGCA	TGCTTCGCTGGTGAAGTTCT
SOCS3	TGGTCACCCACAGCAAGTTC	GACTCCGTCTTGACGCTGAG
Fas	ATTGCGAGCCATGTACCACA	ATGGCATTCCTTCCACTCCG
FADD	GGAAACAGGAACGAAAGCCG	AAAGGAATGCAGCAGGGTCA
Caspase8	CTACCGGTGCCAAGATCACA	ATCACCTTGGCATGCTTCCT
Caspase3	TGCGAGCGTCAGAGAAGTTT	CTTCTGCACTTGTCACCCCT
BAK	GAGAGAGAAGAGAGCGGGGA	TTCAAACAGGCTGGAGGCAA
Cyt C	ATGTTCTCAGTGCCACACAGTT	GTTCTGCGGCCAAATAAACCC
APAF1	ACGAAGTACTAACAACAGAGGAGG	GCCGAGTGACAAACACAACC
Caspase9	CTGCCTACCAGAACCCTACA	AGGATCGAGAGGAACACGGG
β-Actin	GATGATATTGCTGCGCTCGTT	GATGGATGGGAAGACGGCAC

### Western blotting

Cells were washed with PBS and total protein was extracted using RIPA buffer (Beyotime, China, P0013C). Whole-cell lysates were separated by 10% SDS-PAGE, followed by transfer to polyvinylidene difluoride (PVDF) membranes. Membranes were blocked with rapid sealing solution at room temperature for 10 min on a shaker. Primary antibody was incubated overnight at 4 ℃ and secondary antibody was incubated at room temperature for 1 h. Immunoreactive bands were visualized using an electrochemiluminescence (ECL; beyotime, China, P0018S) western blotting reagent. The film was scanned, and the band density was quantified using Image J software.

### Immunofluorescence

Immunofluorescence was performed on DEFs transfected with target gene overexpression vectors. After 4% paraformaldehyde (PFA) fixation, the cells were permeabilized with 0.5% Triton X-100 for 15 min and blocked with 5% BSA for 1 h at room temperature. Then the cells were incubated with primary antibody overnight at 4 °C and secondary antibody was incubated at room temperature away from light for 1 h. DAPI was used to stain nuclei. Confocal microscopy was used for the acquisition and analysis of immunofluorescence staining.

### TUNEL assay for apoptosis

Cell apoptosis induced by H5N6 AIV infection was detected using the TUNEL kit (Beyotime, China, C1086) according to the manufacturer’s instructions. At the appropriate time point after inoculation, cells were first washed once with pre-cooled PBS. Then cells were fixed in 4% PFA for 30 min at room temperature and washed with PBS. After incubating with TUNEL reaction solution for 1 h at 37 °C in the dark, cells were washed twice with PBS. TUNEL-positive cells were visualized by green fluorescence, and images were observed under a fluorescence microscope.

### Mitochondrial membrane potential detection

Mitochondrial membrane potential was assessed using a Mitochondrial Membrane Potential Assay Kit with JC-1 (beyotime, China, C2006), following the manufacturer’s instructions. Briefly, the culture medium was removed from DEFs grown in 6-well plates, and 1 mL of JC-1 working solution was added to each well. The cells were incubated at 37 °C for 20 min. After incubation, the cells were washed twice with 1 × JC-1 Staining Buffer. Finally, 2 mL of PBS was added, and the cells were visualized under a fluorescence microscope.

### Caspase activity assay

Caspase activities were determined with caspase activity assay kits, including Caspase 3 Activity Kit (Beyotime, China, C1115), Caspase 8 Activity Kit (Beyotime, China, C1151) and Caspase 9 Activity Kit (Beyotime, China, C1157). Briefly, treated cells were digested using trypsin, centrifuged at 600 *g* for 5 min at 4 °C and then lysed using lysis buffer. Subsequently, cell lysates were incubated with specific caspase substrates (Ac-DEVD-pNA for caspase3, Ac-IETD-pNA for caspase8, Ac-LEHD-pNA for caspase9, respectively) at 37 °C for 1 h. After incubation, the formation of yellow p-nitroaniline (pNA) was determined at 405 nm using a microplate reader (Bio Tek, USA).

### Flow cytometric analysis of apoptotic cells

After digesting with EDTA-free trypsin and centrifuging, cells were resuspended in PBS and counted. The resuspended cells were centrifuged again to remove the supernatant. Cells were mixed with 195 µL 1 × AnnexinV binding buffer, followed by gentle addition of 5 µL of Annexin V-fluorescein isothiocyanate (V-FITC) and 10 µL of propidium iodide (PI) and incubated at room temperature in the dark for 10–20 min. After staining, the samples were placed in an ice box and assayed for apoptosis using a flow cytometer within 1 h.

### Statistical analysis

Statistical analyses were performed by GraphPad Prism 8 (GraphPad Software Inc., San Diego, CA, USA). All experiments were carried out with a minimum of three parallel samples. The results are presented as mean ± SEM. * *P* < 0.05, **P < 0.01, ***P < 0.001, ns indicates not significant.

## Results

### Systematic identification of duck ISGs

In the present study, we first identified type II IFN activity on the basis of the anti-VSV activity of IFN on DEFs. The suppression of cytopathic effects elicited by both VSV and H5N6 AIV under various concentrations of IFN is delineated in the Additional file 1A-B. Following the assessment of the anti-VSV activity, the bioactivity of the rduIFN-γ protein within DEFs was determined to be 5.9 × 10^3^ units per milligram (U/mg). Subsequently, we established that rDuIFN-γ possesses potent antiviral activity against the H5N6 AIV, with an efficacy of 2.29 × 10^4 U/mL.

**Figure 1:**
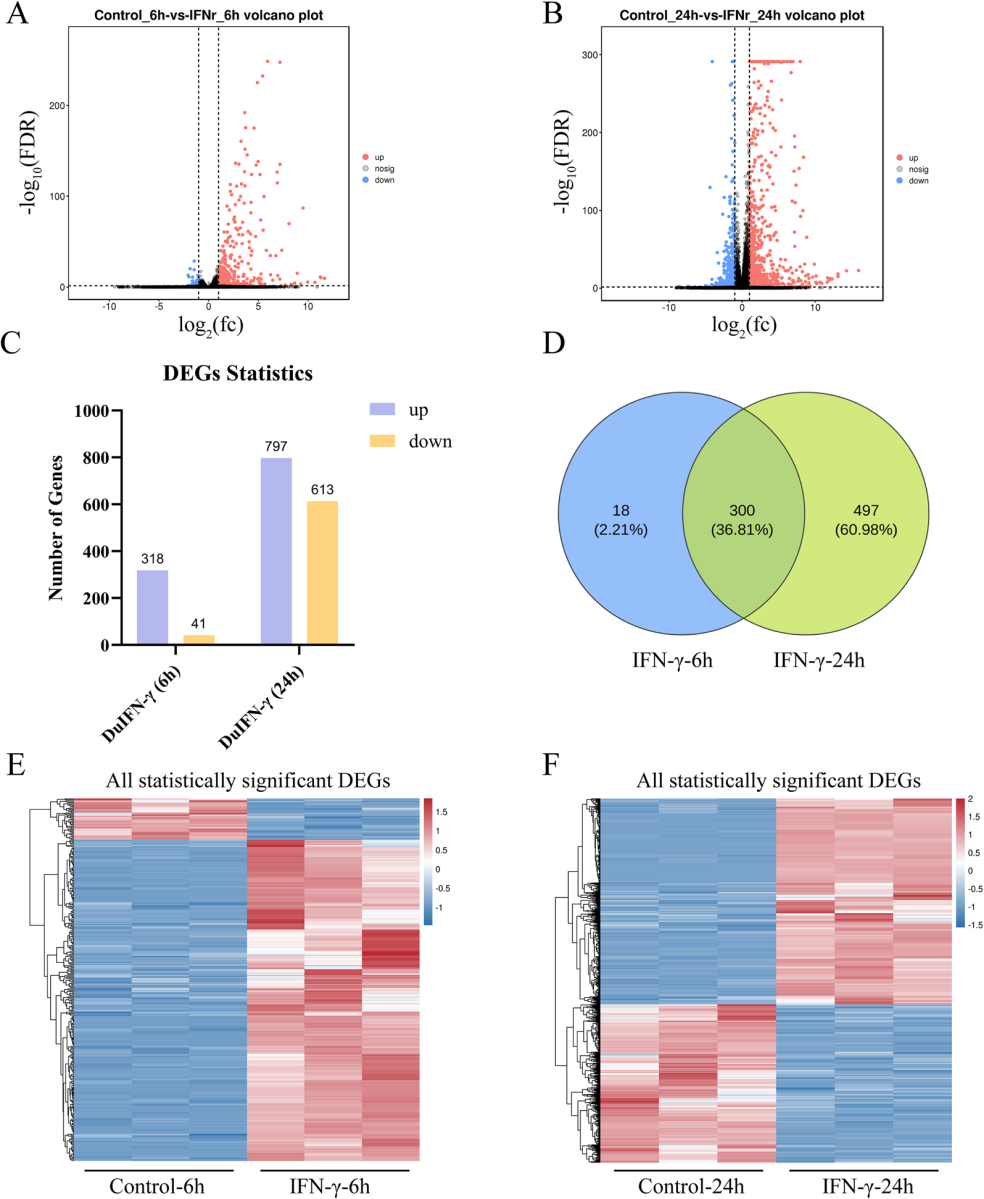
**Systematic identification of duck ISGs.** **A**, **B** Volcano plot of DEGs in DEFs treated with duIFN-γ at 6 h and 24 h post treatment.** C** DEGs in duIFN-γ-stimulated cells were detected by RNA-Seq. **D** Venn diagram illustrating the overlap of upregulated ISGs from duIFN-γ-stimulated DEFs between 6 and 24 h time points. The total number of identified type II ISGs is 815, representing the union of genes upregulated at 6 h (318 genes) and 24 h (797 genes). The intersection (300 genes) indicates ISGs upregulated at both time points. **E** Heatmap displaying the expression profiles of all statistically significant DEGs (including both upregulated and downregulated genes; |log_2_FC|> 1, FDR < 0.05) in DEFs treated with IFN-γ for 6 h versus control. **F** Heatmap displaying the expression profiles of all statistically significant DEGs (including both upregulated and downregulated genes; |log_2_FC|> 1, FDR < 0.05) in DEFs treated with IFN-γ for 24 h versus control. qPCR and RNA-seq results were respectively displayed as 2^−ΔΔCt^ value and the average log_2_ (fold change) values of DEGs. Data are representative of three independent experiments, *P < 0.05, **P < 0.01, ***P < 0.001, ns: no significant difference.

By RNA-Seq, using the criteria of |log_2_FC|> 1 and FDR < 0.05, DEGs were categorized into up-regulated, down-regulated and concertedly regulated genes and presented in a volcano plot (Figures 1A, B). Compared with controls, 318 and 797 genes were upregulated in 6- and 24-h IFN-γ-treated DEFs, respectively. In particular, 300 genes were synchronously upregulated at both time points (Figures 1C, D). We comprehensively identified a total of 815 duck type II ISGs (representing the union of genes upregulated at both time points), and the expression patterns of all significant DEGs were depicted in heatmaps (Figures 1E, F), showing clustering and reproducibility between IFN-treated and untreated samples.

### Enrichment analysis of duck ISGs in DuIFN-induced DEFs

**Figure 2:**
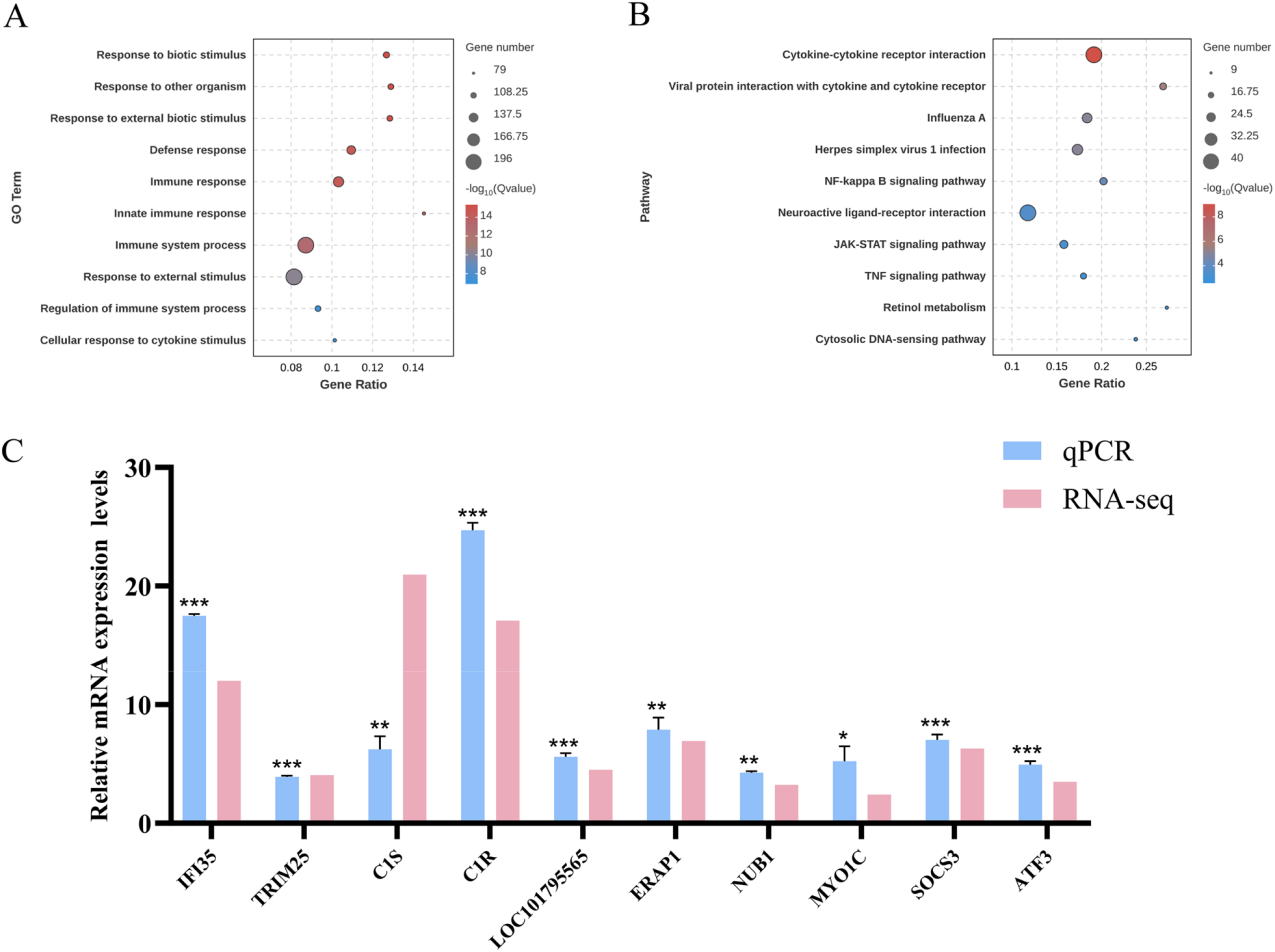
**Enrichment analysis of duck ISGs in DuIFN-induced DEFs.** **A** GO enrichment analysis of duck ISGs at 6 h and 24 h post IFN-γ stimulation. The top 10 significantly enriched GO terms under the “Biological Process” category are shown. Dot size is proportional to the number of duck ISGs, and color represents enrichment significance (-log_10_ q-value). **B** KEGG pathway enrichment analysis of duck ISGs. Significantly enriched pathways include cytokine-cytokine receptor interaction, influenza A, JAK-STAT signaling, and NF-κB signaling. Dot size reflects gene number per pathway, and color indicates statistical significance. **C** Ten ISGs were selected from duIFN-γ-stimulated DEFs for expression validation. Relative mRNA levels were determined by the ratio of the duIFN-treatment group results to the control group results. qPCR and RNA-seq results were respectively calculated as 2^−ΔΔCt^ value and the average log_2_ (fold change) values of duck ISGs. Data are representative of three independent experiments, *P < 0.05, **P < 0.01, ***P < 0.001, ns:no significant. difference

GO enrichment analysis and KEGG pathway analysis were employed to analyze duck ISGs. As illustrated in Figure 2A, the GO enrichment analysis revealed that following IFN treatment, the duck ISGs were predominantly enriched in “response to biotic stimulus,” “defense response,” and “innate immune response.” Moreover, the GO enrichment categories for the duck ISGs included pathways related to the innate immune response. Figure 2B presents the KEGG pathway analysis, indicating that after IFN treatment, pathways such as “cytokine-cytokine receptor interaction,” “viral protein-cytokine-receptor interaction,” and “Influenza A” were significantly activated, suggesting a potential association between duck ISGs and Influenza A.

To validate the authenticity of the RNA-Seq results, we randomly selected 10 duck ISGs and verified their relative expression levels between the control group and the IFN-treated group using qPCR. As depicted in Figure 2C, the expression trends of these selected ISGs were consistent with the RNA-Seq data, thereby affirming their reliability.

### siRNA-mediated knockdown reveals antiviral roles of six duck ISGs

**Figure 3:**
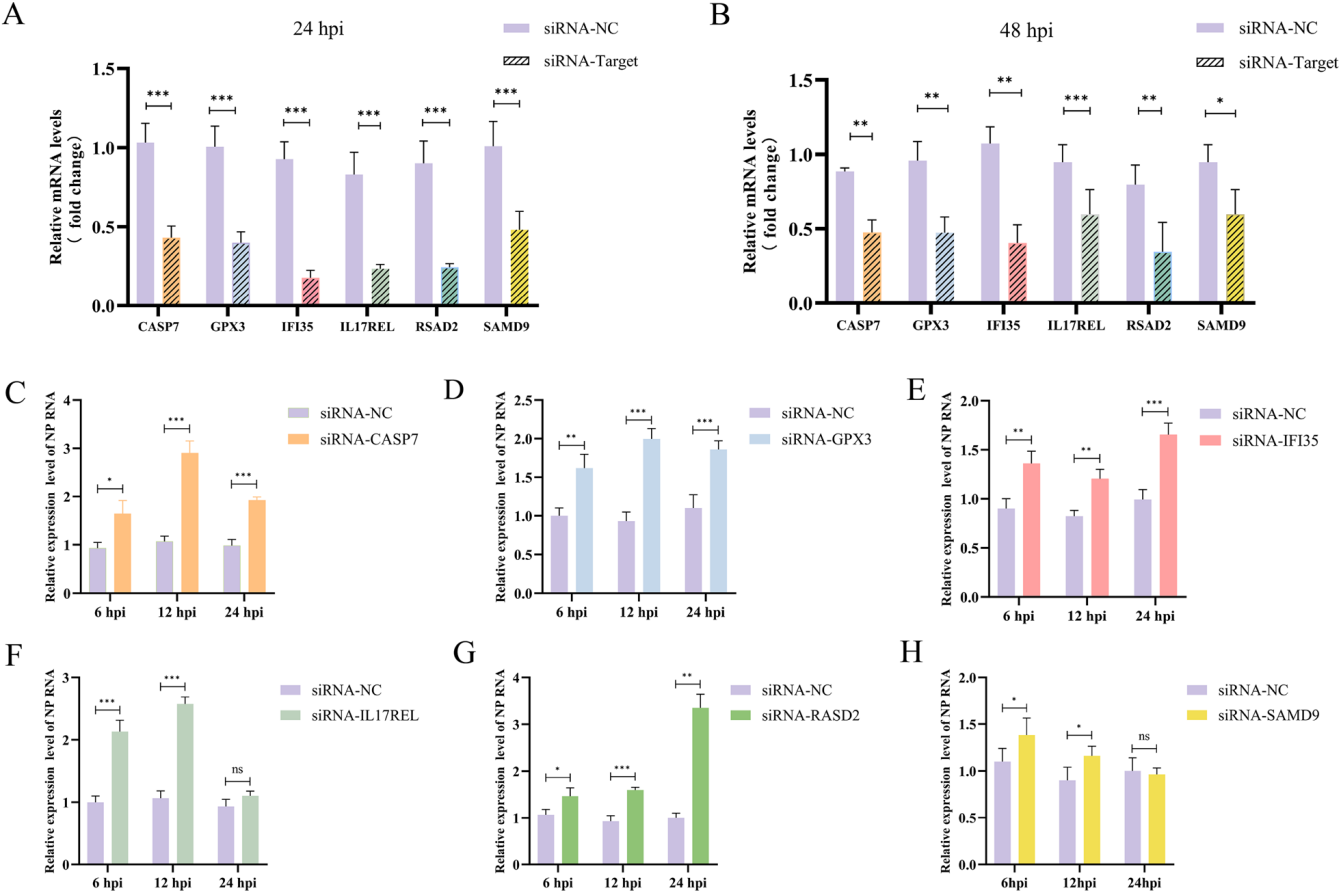
**siRNA-mediated knockdown reveals antiviral roles of six duck ISGs, including** ***duIFI35.*** **A**, **B** RT-qPCR was performed to analyze the mRNA levels of genes encoding *duCASP7, duGPX3, duIFI35, duIL17REL, duRSAD2* and *duSMAD9* in DEFs transfected with siRNA at 24 h and 48 h respectively. **C**–**H** Expression of viral gene NP after knockdown of *duCASP7, duGPX3, duIFI35, duIL17REL, duRSAD2* and *duSMAD9* in DEFs was detected by RT-qPCR at 6 h, 12 h and 24 h after H5N6 AIV infection, respectively. Data are representative of three independent experiments, *P < 0.05, **P < 0.01, ***P < 0.001, ns:no significant difference.

The KEGG pathway analysis delineated a cluster of 25 genes within the Influenza A pathway. Among these genes, six exhibited significant upregulation, and we delved into an in-depth study to elucidate their antiviral properties. In this study, the transfection of siRNA was employed to diminish the mRNA levels of the candidate genes. The specific sequences of all siRNAs used are listed in Table 2. As depicted in Figures 3A, B, the mRNA levels of ISGs in siRNA-transfected cells were markedly reduced compared to control cells. The efficacy of RNA interference was confirmed, demonstrating that the siRNAs targeting the six duck ISGs substantially lowered their expression levels.

**Table 2:** **Sequences of siRNAs used in this study**

SiRNA Name	Sense	Antisense
si-GPX3	GCCACCGUGAAGAACGACATT	UGUCGUUCUUCACGGUGGCTT
si-CASP7	GCCAAAGUCCUCUCUCAGUTT	ACUGAGAGAGGACUUUGGCTT
si-SAMD9	GCAUUAGAGAACCUAGAUUTT	AAUCUAGGUUCUCUAAUGCTT
si-IL17REL	GCUUGUCACGGUUAACCUATT	UAGGUUAACCGUGACAAGCTT
si-RASD2	GCCGAGUGUCAGCAUCGUUTT	AACGAUGCUGACACUCGGCTT
si-IFI35	GGAUACAACAGGUGAAGAATT	UUCUUCACCUGUUGUAUCCTT

To evaluate the antiviral effects of the candidate genes, we meticulously examined the transcriptional profile of the viral nucleoprotein (NP) protein. Following RNA interference against *duCASP7*, *duGPX3*, *duIFI35*, *duI17REL*, *duRASD2*, and *duSAMD9*, the RNA levels of the NP protein in H5N6 AIV-infected groups were significantly higher than those in the control groups (Figures 3C–H). The findings suggest that the selected ISGs effectively inhibit the replication of NP in H5N6 AIV. Additional file 2–7 respectively record the phylogenetic trees constructed based on the amino acid sequences of ducks and other species for these six genes. Among the six ISGs with antiviral activity, we selected *duIFI35* for further exploration of its antiviral effects.

### H5N6 AIV infection activates both intrinsic and extrinsic apoptosis pathways in DEFs

Following the identification of *duIFI35* as an antiviral ISG, we aimed to elucidate its mechanism of action. Prior studies have shown that certain ISGs exert their antiviral functions by modulating programmed cell death pathways such as apoptosis and pyroptosis, thereby limiting viral replication and dissemination [16, 17]. Moreover, *IFI35* has been previously implicated in apoptosis regulation in both viral and inflammatory contexts [18, 19]. Given this evidence, we hypothesized that the antiviral activity of *duIFI35* might be linked to its potential role in promoting apoptosis during H5N6 infection.

**Figure 4:**
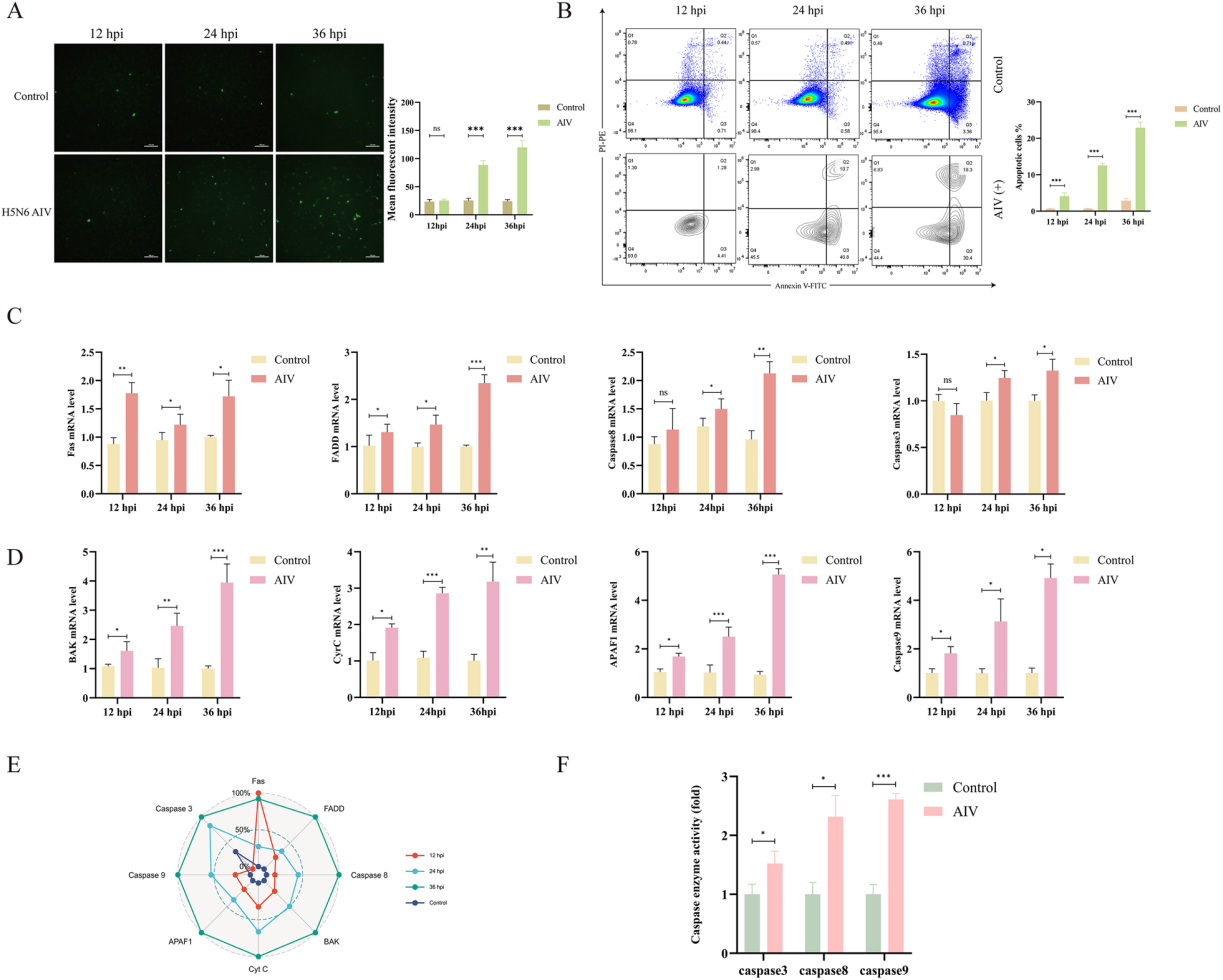
**H5N6 AIV infection activates both intrinsic and extrinsic apoptosis pathways in DEFs**. **A** TUNEL assay was used to detect apoptosis in DEFs at 12, 24, and 36 hpi with H5N6 AIV. Green fluorescence indicates TUNEL-positive apoptotic cells. The bar graph shows the quantified fluorescence intensity. Scale bar = 100 μm. **B** Flow cytometry analysis of apoptosis in DEFs using Annexin V-FITC and PI staining at indicated time points post-infection. The percentage of apoptotic cells significantly increased in H5N6 AIV-infected groups over time. **C** mRNA levels of extrinsic apoptotic markers (*Fas, FADD, caspase-8, and caspase-3*) were assessed by RT-qPCR at 12, 24, and 36 hpi. **D** mRNA levels of intrinsic apoptotic pathway-related genes (*BAK, cytochrome c, APAF1, and caspase-9*) in infected versus control groups at the same time points. **E** The radar chart depicting the expression of apoptosis-related genes. **F** Enzymatic activities of *caspase-3*, *caspase-8*, and *caspase-9* were measured at 24 hpi, confirming the activation of both apoptotic pathways. Data are representative of three independent experiments, *P < 0.05, **P < 0.01, ***P < 0.001, ns:no significant difference.

To explore this, we first sought to determine whether H5N6 AIV infection alone is capable of inducing apoptosis in DEFs. Apoptosis was evaluated using TUNEL staining and Annexin V/PI flow cytometry at different time points post-infection. H5N6 AIV infection induced apoptosis in DEFs in a time-dependent manner. Apoptosis was detectable at 12 hpi, significantly increased at 24 hpi, and peaked at 36 hpi, as shown by both fluorescence imaging and flow cytometry (*P* < 0.001, Figures 4A, B). To further characterize the apoptosis signaling pathways involved, we analyzed the expression of genes associated with both extrinsic and intrinsic apoptotic pathways. qPCR revealed significant upregulation of *Fas*, *FADD*, and *Caspase-8*, which are key mediators of the extrinsic death receptor pathway, as well as *BAK*, *cytochrome c*, *APAF1*, and *Caspase-9*, markers of intrinsic apoptosis (Figures 4C–E). These changes were accompanied by an increase in Caspase-3, -8, and -9 enzymatic activity (Figure 4F), confirming the activation of both types of apoptosis activation.

### duIFI35 suppresses H5N6 replication in DEFs

**Figure 5:**
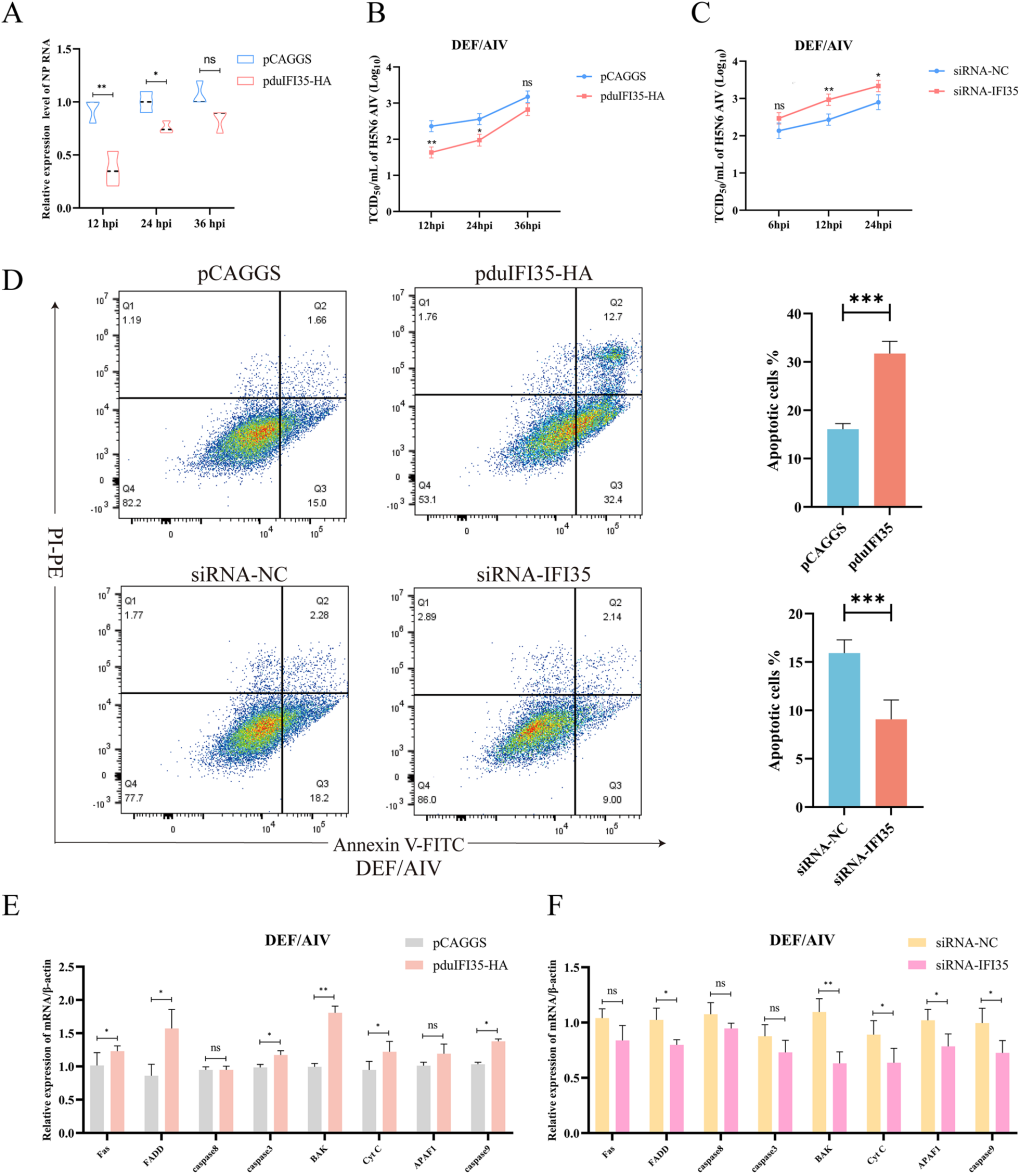
**duIFI35 erexpression negatively correlates with H5N6 replication in DEFs**. **A** Relative mRNA levels of H5N6 NP gene in DEFs transfected with either pCAGGS empty vector or pduIFI35-HA at 12, 24, and 36 hpi, measured by RT-qPCR. **B** Virus titers of H5N6 AIV in DEFs overexpressing *duIFI35* (pduIFI35-HA) compared to control (pCAGGS) at indicated time points, determined by TCID_50_ assay. **C** H5N6 viral titers after knockdown of *duIFI35* using siRNA in DEFs compared to siRNA-negative control. **D** Flow cytometry analysis of apoptosis in DEFs transfected with *duIFI35*-HA (upper panel) or siRNA-*IFI35* (lower panel) and infected with H5N6 AIV. Quantification of apoptotic cell percentages is shown in the right bar graphs. **E** Relative expression levels of apoptosis-related genes (*Fas, FADD, caspase-8, caspase-3, BAK, Cyt-c, APAF1, caspase-9*) in DEFs overexpressing *duIFI35* versus vector control, measured by RT-qPCR. **F** mRNA levels of the apoptosis-related genes (*Fas, FADD, caspase-8, caspase-3, BAK, Cyt-c, APAF1, caspase-9*) in DEFs transfected with siRNA-*IFI35* versus siRNA-NC. Data are representative of three independent experiments, *P < 0.05, **P < 0.01, ***P < 0.001, ns:no significant difference.

Given that *IFI35* has previously been reported to regulate cell death pathways, we investigated whether *duIFI35* exerts its antiviral function by promoting apoptosis of DEFs during H5N6 AIV infection. DEFs were transfected with the pduIFI35-HA plasmid and subsequently infected with H5N6 AIV. Using an HA-tag specific antibody, immunofluorescence analysis confirmed the successful overexpression of *duIFI35*. Furthermore, co-staining with the mitochondrial outer membrane marker TOMM20 revealed that the duIFI35-HA protein did not colocalize with mitochondria (Additional file 8). Subsequent assessment of viral replication revealed that overexpression of *duIFI35* significantly inhibited viral proliferation at early stages of infection. RT-qPCR analysis showed that mRNA levels of the viral NP gene were significantly reduced at 12 and 24 h post-infection (Figure 5A). Consistently, TCID_50_ assays indicated a marked decrease in viral titers at the same time points (Figure 5B). Conversely, knockdown of *duIFI35* increased H5N6 viral titers in DEFs. Compared to siRNA-NC, siRNA-*IFI35* led to significantly higher titers at 12 hpi (*P* < 0.01) and 24 hpi (*P* < 0.05), but not at 6 hpi (Figure 5C). We next examined whether *duIFI35* mediates its antiviral effects by regulating apoptosis. The gating strategy for assessing apoptosis by flow cytometry is shown in Additional file 9. The results of flow cytometry revealed that overexpression of *duIFI35* promoted apoptosis in DEFs 24 h post-H5N6 infection, whereas siRNA-mediated knockdown inhibited apoptosis (Figure 5D). Similarly, transcription of apoptosis-related genes was significantly upregulated upon *duIFI35* overexpression and downregulated upon its knockdown (Figures 5E, F). Finally, we assessed changes in mitochondrial membrane potential using the JC-1 probe. Additional file 10 demonstrates that duIFI35 overexpression led to a more rapid and severe reduction in mitochondrial membrane potential than that observed in the vector control at 12, 24, and 36 hpi.

These findings indicate that *duIFI35* promotes apoptosis of DEFs during H5N6 AIV infection, suggesting that its antiviral activity may be mediated through the induction of programmed cell death.

### Apoptosis is required for the antiviral activity of duIFI35

To further verify whether the antiviral effect of *duIFI35* is apoptosis-dependent, we utilized the pan-caspase inhibitor Z-VAD-FMK (20 μM, Beyotime, C1202) to block apoptosis and evaluated its impact on viral replication in the presence or absence of *duIFI35* overexpression.

**Figure 6:**
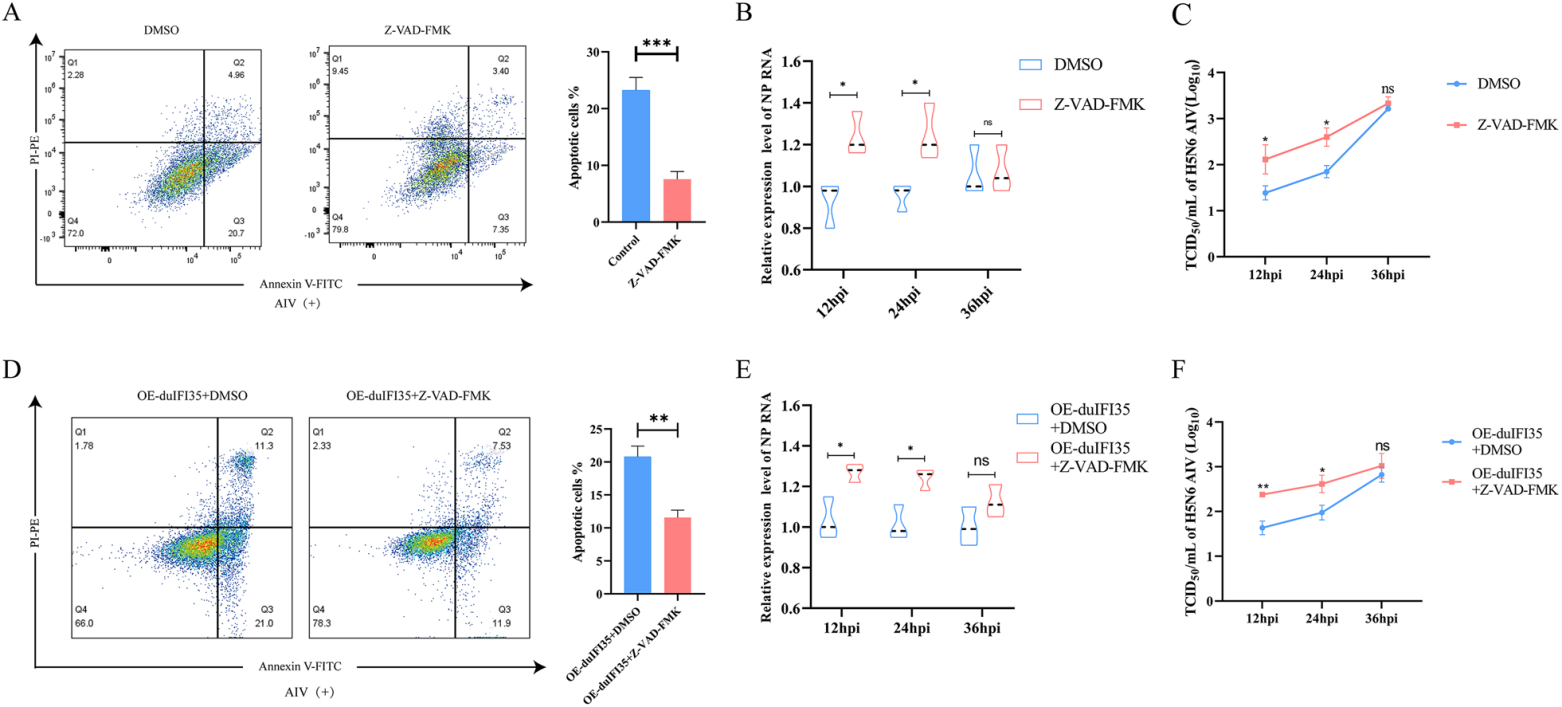
**Apoptosis is required for the antiviral activity of *duIFI35.*** **A** Flow cytometry analysis of apoptosis in H5N6-infected DEFs treated with pan-caspase inhibitor Z-VAD-FMK or DMSO control. Bar graph shows percentage of apoptotic cells. **B** Relative NP RNA levels in DEFs treated with Z-VAD-FMK or DMSO, measured by RT-qPCR. **C** Viral titers of H5N6 AIV in DEFs treated with Z-VAD-FMK or DMSO, measured by TCID_50_ assay at the indicated time points. **D** Flow cytometry analysis of apoptosis in DEFs overexpressing duIFI35 (OE-*duIFI35*) with or without Z-VAD-FMK treatment. Right panel quantifies apoptotic cell percentages. **E** NP RNA levels in OE-*duIFI35* cells treated with DMSO or Z-VAD-FMK. **F** TCID_50_-based viral titers in OE-*duIFI35* cells treated with or without Z-VAD-FMK. Data are representative of three independent experiments, *P < 0.05, **P < 0.01, ***P < 0.001, ns:no significant difference.

Flow cytometry analysis showed that Z-VAD-FMK treatment significantly reduced the percentage of apoptotic cells compared with the untreated virus-infected group (Figure 6A), confirming that H5N6-induced apoptosis was successfully inhibited. Subsequently, we measured viral replication levels by RT-qPCR of the NP gene and TCID_50_ assay. As shown in Figure 6B, C, Z-VAD-FMK treatment led to a significant increase in both viral RNA levels and infectious viral titers at 24 h post-infection. These results suggest that apoptosis serves as a host defense mechanism that restricts H5N6 viral replication by limiting viral propagation.

To further determine whether the antiviral effect of *duIFI35* is mechanistically dependent on apoptosis, DEFs were transfected with pduIFI35-HA and infected with H5N6 virus in the presence or absence of Z-VAD-FMK. Flow cytometry analysis revealed that the apoptosis-promoting effect of *duIFI35* was suppressed upon Z-VAD-FMK treatment (Figure 6D). Interestingly, when apoptosis was inhibited, NP gene expression levels increased significantly (Figure 6E). Consistently, TCID_50_ assay results showed that viral titers were also elevated following Z-VAD-FMK treatment (Figure 6F). Together, these findings suggest that *duIFI35* suppresses H5N6 replication via apoptosis induction, and that apoptosis is essential for its antiviral function.

## Discussion

IFN-mediated immune responses can trigger antiviral mechanisms at the cellular level and are a key component of the immune defense network. Previous studies have demonstrated that H5Nx AIV is particularly susceptible to IFN-γ, an effect closely linked to the induction of ISGs [3, 10]. In previous studies, ISGs in the transcriptomes of various cell types have been identified, but avian ISGs have not been fully explored. Therefore, in-depth research on the specific mechanisms by which ISGs inhibit avian influenza viruses will help to fully understand the innate immune response of the host against avian influenza viruses and may provide new ideas for the effective control of these viruses. Although a large number of ISGs have been identified in mammals, research on avian ISGs is relatively lagging. In our preliminary study, type I, II, and III IFNs in chicken have been identified [20]. As natural reservoirs of avian influenza viruses, ducks play a pivotal role in understanding host resistance mechanisms. Type I and III IFNs are well-known for their roles in antiviral defense, particularly during early infection and in mucosal immunity [21, 22]. Their downstream ISG responses in ducks have been more extensively studied. Research by Zhou et al. indicated that both duIFN-β and duIFN-λ can induce the expression of duck ISGs and exhibit significant antiviral activity against Duck Tembusu virus in vitro and in vivo [23]. Another study has also shown that duSTING (stimulator of interferon genes) is essential for type I interferon induction in ducks and plays a critical role in host defense against duck plague virus infection [24]. In contrast, the functionality and specific ISG profiles induced by type II IFN in ducks remain largely unexplored. In this study, we conducted a systematic identification of duck type II ISGs.

Previous effort has employed multiple transcriptomic approaches to identify type I ISGs in primary chicken embryo fibroblasts [25]. Zhou et al. described the cross-species antiviral activity of goose IFN-α and IFN-γ against duck plague virus [26]. Despite these advances, a comprehensive identification of duck ISGs, particularly those induced by type II interferon, remains lacking [27]. In this study, we systematically identified type II ISGs derived from DEFs using RNA-seq, which generated 1441 statistically significant DEGs, with 815 upregulated and 626 downregulated. There are many methods for screening antiviral ISGs, and overexpression or siRNA screening has been widely used. After siRNA screening in this study, six genes were obtained: glutathione peroxidase 3 (*GPX3*), interleukin 17 receptor E like (*IL17REL*), radical S-adenosyl methionine domain containing 2 (*RSAD2*), caspase 7 (*CASP7*), sterile alpha motif domain-containing protein 9 (*SAMD9*), and IFN induced protein 35 (*IFI35*). Preliminary validation showed that all six genes have antiviral activity.

*GPX3* (XM_027468347.3) is a unique extracellular selenoprotein belonging to the reductase family, whose main function is to catalyze the detoxification of water-soluble lipid hydroperoxides by reduced glutathione [28]. Although our results confirmed the antiviral activity of *GPX3*, its precise mechanism of action remains to be fully elucidated. *IL17REL* (XM_038173189.2) may be a new member of the IL17R family. Although research on it in ducks is relatively limited, it is generally believed that it plays a similar role in inflammatory responses as other *IL17R* members [29]. *RSAD2* (NM_001310801.1), also known as the IFN-induced endoplasmic reticulum-associated antiviral gene, is a multifunctional IFN-stimulated protein that has been reported to exhibit broad-spectrum antiviral activity against various RNA viruses, including influenza virus, flaviviruses, and hepatitis C virus (HCV) [30]. *CASP7* (XM_027459864.3) is an execution caspase with a role in apoptosis. A study pointed out that it can activate a protein called acid sphingomyelinase, which initiates the cell membrane repair mechanism, allowing the cell to complete its final cellular processes before death [31]. *SAMD9* (XM_005010927.6) and SAMD9L are homologous genes with both antiviral and anti-tumor functions and play an important role in the host’s innate immune response [32]. A study has shown that *IFI35* (XM_038168772.2) has significant antiviral activity, including against Newcastle disease virus, foot-and-mouth disease virus, bovine foamy virus, and prototype foamy virus [33]. This study has preliminarily confirmed at the RNA level that these six genes inhibit H5N6 AIV.

According to current reports, AIV infection triggers multiple programmed cell death pathways in host cells. Among these, apoptosis, as a host defense mechanism, plays a critical role in combating viral infections. To counter viral infection, host cells often trigger apoptosis to remove infected counterparts and suppress viral replication and dissemination. However, many viruses have evolved mechanisms to exploit host apoptotic pathways to facilitate their propagation. For instance, Newcastle disease virus exploits “apoptotic mimicry” by acquiring host phosphatidylserine during budding to enhance viral entry and infectivity [34]. Fowl adenovirus serotype 4 facilitates its own replication by inducing a host microRNA that targets MCL-1 to trigger apoptosis [35]. Conversely, our results indicate that in the context of H5N6 infection, apoptosis functions not as a viral tool, but as a host defense mechanism to curtail viral spread. Furthermore, some studies suggest that influenza viruses may engage various cell death pathways, including intrinsic apoptosis (mitochondrial pathway), extrinsic apoptosis (death receptor pathway), and necrosis [36–38]. However, the molecular mechanisms by which influenza viruses induce apoptosis remain incompletely understood. Our study found that H5N6 AIV infection in DEFs significantly upregulated key factors associated with both intrinsic and extrinsic apoptosis. Notably, inhibiting apoptosis increased viral NP replication, indicating that H5N6 AIV triggers apoptotic mechanisms in DEFs to limit viral replication.

Apoptosis is a regulated, programmed cell death that may act like a homeostatic control of cell growth or a cellular immune response to invading pathogens [39]. Current research on apoptosis increasingly emphasizes its interplay with immune signaling pathways, particularly in the context of antiviral defense. Among the six antiviral genes that were screened, *IFI35* attracted our attention because of its role in innate immunity, leading us to investigate how it exerts its function. A recent study indicated that targeted inhibition of *IFI35* suppresses apoptosis via the JAK1/STAT1 signaling pathway [18]. Additionally, Hu et al. reported that upregulation of *IFI35* promotes apoptosis, whereas its downregulation inhibits it [40]. *IFI35* is widely transcribed in various cell lines, including fibroblasts and mononuclear macrophages, and its expression is significantly enhanced under IFN stimulation, highlighting its critical role in host antiviral responses [41]. Our findings show that inhibition of early apoptosis led to enhanced viral replication. Additionally, knocking down *duIFI35* inhibited apoptosis induced by AIV, resulting in a sharp increase in viral NP RNA levels. Conversely, overexpression of *duIFI35* promoted apoptosis, leading to a significant decrease in viral NP RNA levels. These findings suggest that the inhibitory effect of *duIFI35* on H5N6 AIV is likely achieved through the regulation of apoptosis. Although our study was conducted in vitro, the physiological relevance of *IFI35* is established in other models. Research indicates that *IFI35* expression is significantly upregulated in various tissues of chickens upon Newcastle disease virus infection [33]. Given avian influenza virus characteristics, we propose that *duIFI35* likely restricts viral spread in key tissues like respiratory and intestinal epithelia in live ducks. Identifying *duIFI35* as a pro-apoptotic factor clarifies duck disease tolerance. Unlike chickens, which succumb to cytokine storms, ducks utilize *duIFI35*-mediated apoptosis as a “clean” clearance mechanism. This strategy limits viral spread while minimizing inflammation, effectively balancing viral clearance with tissue protection to prevent the fatal immunopathology common in susceptible poultry.

In summary, this study employed RNA-Seq technology to preliminarily screen 815 duck type II ISGs. We successfully validated that six genes, including *duIFI35*, possess the ability to inhibit H5N6 AIV replication. Ultimately, through multi-faceted analysis, we demonstrated that one of the antiviral effects of *duIFI35* is to block viral replication by inducing apoptosis in infected cells.

## Supplementary Information

**Additional file 1** **Activity detection of recombinant DuIFN-γ against vesicular stomatitis virus (VSV) and H5N6 AIV in vitro.** (A) Cytopathic effects (CPE) caused by VSV (100 ¬TCID50) in DEFs preincubated with different concentrations of recombinant DuIFN-γ. Dilution factor (only the CPE results of two dilution factors at the critical point are shown): 4−5 (no CPE), 4−6 (CPE appearance). (B) CPE induced by H5N6 AIV in DEFs preincubated with different concentrations of recombinant DuIFN-γ. Dilution factor 10-3(no CPE), 10-4(CPE appearance). Abbreviations: NC, negative control (mock treated cells); VSV-GFP+, positive control (cells are directly inoculated with VSV without IFN treatment); H5N6 AIV+, positive control (cells are directly inoculated with H5N6 AIV without IFN treatment).

**Additional file 2** **Phylogenetic tree constructed based on amino acid sequences of duCASP7 (XM_027459864.3) and CASP7 from other species**.

**Additional file 3** **Phylogenetic tree constructed based on amino acid sequences of duGPX3 (XM_027468347.3) and GPX3 from other species.**

**Additional file 4** **Phylogenetic tree constructed based on amino acid sequences of duIFI35 (XM_038168772.2) and IFI35 from other species.**

**Additional file 5** **Phylogenetic tree constructed based on amino acid sequences of duIL17REL (XM_038173189.2) and IL17REL from other species.**

**Additional file 6** **Phylogenetic tree constructed based on amino acid sequences of duRSAD2 (NM_001310801.1) and RSAD2 from other species**.

**Additional file 7** **Phylogenetic tree constructed based on amino acid sequences of duSMAD9 (XM_005010927.6) and SMAD9 from other species.**

**Additional file 8** **Expression of duIFI35 in DEFs.** (A)WoLF PSORT II predicted the subcellular localization of duIFI35, showing its distribution (in blue) primarily in the cytoplasm. (B) Expression of pduIFI35-HA. (C) Subcellular localization of duIFI35 in DEFs. Red: anti-HA; Green: anti-TOMM20 (mitochondria); Blue: DAPI (nuclei). Scale bar, 20 μm.

**Additional file 9** **The gating strategy of apoptotic cells in DEFs. **(A) Gating strategy of DEFs living cells. (B) Gating strategy of DEFs without adhesions. (C) Gating strategy of DEFs apoptotic cells. Q1, Q2 are dead cells, Q3 are apoptotic cells, and Q4 are living cells.

**Additional file 10** **Overexpression of duIFI35 induces time-dependent mitochondrial depolarization in H5N6-infected DEFs.** DEFs were transfected with empty vector or pCAGGS-duIFI35 for 24 h and subsequently infected with H5N6 virus. Mitochondrial membrane potential was assessed using JC-1 staining at (A) 12 h, (B) 24 h, and (C) 36 hpi.

## Data Availability

The datasets generated during and/or analysed during the current study are available in the Figshare repository, 10.6084/m9.figshare.30451406 [42].

## References

[1] Wille M, Barr IG (2022) Resurgence of avian influenza virus. Science 376:459–460 35471045 10.1126/science.abo1232

[2] Adlhoch C, Fusaro A, Gonzales JL, Kuiken T, Marangon S, Mirinaviciute G, Niqueux É, Stahl K, Staubach C, Terregino C, Broglia A, Baldinelli F (2023) Avian influenza overview December 2022 – March 2023. EFSA J 21:e7917 10.2903/j.efsa.2023.7917 PMC10025949 36949860

[3] Fleming-Canepa X, Aldridge JRJ, Canniff L, Kobewka M, Jax E, Webster RG, Magor KE (2019) Duck innate immune responses to high and low pathogenicity H5 avian influenza viruses. Vet Microbiol 228:101–111 30593354 10.1016/j.vetmic.2018.11.018 PMC6365012

[4] Zhang B, Xu S, Liu M, Wei Y, Wang Q, Shen W, Lei C, Zhu Q (2023) The nucleoprotein of influenza A virus inhibits the innate immune response by inducing mitophagy. Autophagy 19:1916–1933 36588386 10.1080/15548627.2022.2162798 PMC10283423

[5] Zhao Y, Huang F, Zou Z, Bi Y, Yang Y, Zhang C, Liu Q, Shang D, Yan Y, Ju X, Mei S, Xie P, Li X, Tian M, Tan S, Lu H, Han Z, Liu K, Zhang Y, Liang J, Liang Z, Zhang Q, Chang J, Liu WJ, Feng C, Li T, Zhang MQ, Wang X, Gao GF, Liu Y, Jin N, Jiang C (2022) Avian influenza viruses suppress innate immunity by inducing trans-transcriptional readthrough via SSU72. Cell Mol Immunol 19:702–714 35332300 10.1038/s41423-022-00843-8 PMC9151799

[6] Boehmer D, Zanoni I (2025) Interferons in health and disease. Cell 188:4480–4504 40845809 10.1016/j.cell.2025.06.044 PMC12380125

[7] Schneider WM, Chevillotte MD, Rice CM (2014) Interferon-stimulated genes: a complex web of host defenses. Annu Rev Immunol 32:513–545 24555472 10.1146/annurev-immunol-032713-120231 PMC4313732

[8] Vu TH, Hong Y, Heo J, Kang S, Lillehoj HS, Hong YH (2023) Chicken miR-148a-3p regulates immune responses against AIV by targeting the MAPK signalling pathway and IFN-gamma. Vet Res 54:110 37993949 10.1186/s13567-023-01240-3 PMC10664352

[9] Yang X, Arslan M, Liu X, Song H, Du M, Li Y, Zhang Z (2020) IFN-gamma establishes interferon-stimulated gene-mediated antiviral state against Newcastle disease virus in chicken fibroblasts. Acta Biochim Biophys Sin (Shanghai) 52:268–280 32047904 10.1093/abbs/gmz158 PMC7109688

[10] Gao P, Fan L, Du H, Xiang B, Li Y, Sun M, Kang Y, Chen L, Xu C, Li Y, Ren T (2018) Recombinant duck interferon gamma inhibits H5N1 influenza virus replication in vitro and in vivo. J Interferon Cytokine Res 38:290–297 30016179 10.1089/jir.2018.0034

[11] Malik G, Zhou Y (2020) Innate immune sensing of Influenza A Virus. Viruses 12:755 32674269 10.3390/v12070755 PMC7411791

[12] Song H, Liu X, Gao X, Li J, Shang Y, Gao W, Li Y, Zhang Z (2022) Transcriptome analysis of pre-immune state induced by interferon gamma inhibiting the replication of H(9)N(2) avian influenza viruses in chicken embryo fibroblasts. Infect Genet Evol 103:105332 35811034 10.1016/j.meegid.2022.105332

[13] Kim TH, Zhou H (2018) Overexpression of chicken IRF7 increased viral replication and programmed cell death to the avian influenza virus infection through TGF-Beta/FoxO signaling axis in DF-1. Front Genet 9:415 30356848 10.3389/fgene.2018.00415 PMC6190866

[14] Liu Q, Zhang M, Wang J, Zhang J, Wang Z, Ma J, Yan Y, Sun J, Cheng Y (2022) Functional characterization of bat IRF1 in IFN induction. Dev Comp Immunol 136:104500 35933044 10.1016/j.dci.2022.104500

[15] Thakur V (2024) RNA-Seq data analysis for differential gene expression using HISAT2-StringTie-Ballgown pipeline. Methods Mol Biol 2812:101–113 39068358 10.1007/978-1-0716-3886-6_5

[16] Lu M, Liao F (2011) Interferon-stimulated gene ISG12b2 is localized to the inner mitochondrial membrane and mediates virus-induced cell death. Cell Death Differ 18:925–936 21151029 10.1038/cdd.2010.160 PMC3131945

[17] Zhao L, Li L, Xue M, Liu X, Jiang C, Wang W, Tang L, Feng L, Liu P (2021) Gasdermin D inhibits coronavirus infection by promoting the noncanonical secretion of beta interferon. mBio 13:e360021 10.1128/mbio.03600-21 PMC8805026 35100869

[18] Zhang L, Jiang S, Guo X, Xiao B, Li Q, Chen J, Huang J, Rao H (2021) MiR-146b-5p targets IFI35 to inhibit inflammatory response and apoptosis via JAK1/STAT1 signalling in lipopolysaccharide-induced glomerular cells. Autoimmunity 54:430–438 34435525 10.1080/08916934.2020.1864730

[19] Zheng W, Li X, Wang J, Li X, Cao H, Wang Y, Zeng Q, Zheng SJ (2014) A critical role of interferon-induced protein IFP35 in the type I interferon response in cells induced by foot-and-mouth disease virus (FMDV) protein 2C. Arch Virol 159:2925–2935 25085622 10.1007/s00705-014-2147-7

[20] Dai M, Xie T, Liao M, Zhang X, Feng M (2020) Systematic identification of chicken type I, II and III interferon-stimulated genes. Vet Res 51:70 32448397 10.1186/s13567-020-00793-x PMC7245633

[21] Graves C, Babikow E, Ghaltakhchyan N, Ngo TQ, Liu C, Wang S, Shoji A, Bocklage C, Phillips ST, Markovetz M, Frazier-Bowers SA, Divaris K, Freire M, Wallet S, Wu D, Jacox LA (2024) Immune dysregulation in the oral cavity during early SARS-CoV-2 infection. J Dent Res 103:1258–1270 39394771 10.1177/00220345241271943 PMC11562286

[22] Wimmers F, Burrell AR, Feng Y, Zheng H, Arunachalam PS, Hu M, Spranger S, Nyhoff LE, Joshi D, Trisal M, Awasthi M, Bellusci L, Ashraf U, Kowli S, Konvinse KC, Yang E, Blanco M, Pellegrini K, Tharp G, Hagan T, Chinthrajah RS, Nguyen TT, Grifoni A, Sette A, Nadeau KC, Haslam DB, Bosinger SE, Wrammert J, Maecker HT, Utz PJ, Wang TT, Khurana S, Khatri P, Staat MA, Pulendran B (2023) Multi-omics analysis of mucosal and systemic immunity to SARS-CoV-2 after birth. Cell 186:4632–4651 37776858 10.1016/j.cell.2023.08.044 PMC10724861

[23] Zhou P, Liu D, Zhang Q, Wu W, Chen D, Luo R (2023) Antiviral effects of duck type I and type III interferons against duck Tembusu virus in vitro and in vivo. Vet Microbiol 287:109889 37913673 10.1016/j.vetmic.2023.109889

[24] Chen S, Wu Z, Zhang J, Wang M, Jia R, Zhu D, Liu M, Sun K, Yang Q, Wu Y, Zhao X, Cheng A (2018) Duck stimulator of interferon genes plays an important role in host anti-duck plague virus infection through an IFN-dependent signalling pathway. Cytokine 102:191–199 28969942 10.1016/j.cyto.2017.09.008

[25] Giotis ES, Robey RC, Skinner NG, Tomlinson CD, Goodbourn S, Skinner MA (2016) Chicken interferome: avian interferon-stimulated genes identified by microarray and RNA-seq of primary chick embryo fibroblasts treated with a chicken type I interferon (IFN-alpha). Vet Res 47:75 27494935 10.1186/s13567-016-0363-8 PMC4974698

[26] Zhou H, Chen S, Zhou Q, Wei Y, Wang M, Jia R, Zhu D, Liu M, Liu F, Yang Q, Wu Y, Sun K, Chen X, Cheng A (2016) Cross-species antiviral activity of goose interferons against duck plague virus is related to its positive self-feedback regulation and subsequent interferon stimulated genes induction. Viruses 8:195 27438848 10.3390/v8070195 PMC4974530

[27] Wang X, Li Y, Li L, Shen L, Zhang L, Yu J, Luo Y, Sun Y, Li S, Qiu H (2016) RNA interference screening of interferon-stimulated genes with antiviral activities against classical swine fever virus using a reporter virus. Antiviral Res 128:49–56 26868874 10.1016/j.antiviral.2016.02.001

[28] Chang C, Worley BL, Phaeton R, Hempel N (2020) Extracellular glutathione peroxidase GPx3 and its role in cancer. Cancers (Basel) 12:2197 32781581 10.3390/cancers12082197 PMC7464599

[29] Wu B, Jin M, Zhang Y, Wei T, Bai Z (2011) Evolution of the IL17 receptor family in chordates: a new subfamily IL17REL. Immunogenetics 63:835–845 21732179 10.1007/s00251-011-0554-4

[30] Ebrahimi KH (2018) A unifying view of the broad-spectrum antiviral activity of RSAD2 (viperin) based on its radical-SAM chemistry. Metallomics 10:539–552 29568838 10.1039/C7MT00341B

[31] Nozaki K, Maltez VI, Rayamajhi M, Tubbs AL, Mitchell JE, Lacey CA, Harvest CK, Li L, Nash WT, Larson HN, McGlaughon BD, Moorman NJ, Brown MG, Whitmire JK, Miao EA (2022) Caspase-7 activates ASM to repair gasdermin and perforin pores. Nature 606:960–967 35705808 10.1038/s41586-022-04825-8 PMC9247046

[32] Peng S, Meng X, Zhang F, Pathak PK, Chaturvedi J, Coronado J, Morales M, Mao Y, Qian S, Deng J, Xiang Y (2022) Structure and function of an effector domain in antiviral factors and tumor suppressors SAMD9 and SAMD9L. Proc Natl Acad Sci USA 119:e2116550119 35046037 10.1073/pnas.2116550119 PMC8795524

[33] Jia YQ, Wang XW, Chen X, Qiu XX, Wang XL, Yang ZQ (2022) Characterization of chicken IFI35 and its antiviral activity against Newcastle disease virus. J Vet Med Sci 84:473–483 35135934 10.1292/jvms.21-0410 PMC8983280

[34] Hou Y, Tan L, Zhang P, Zhang D, Chen L, Qiu X, Sun Y, Song C, Liao Y, Ren T, Ding C (2025) Newcastle disease virus acquires phosphatidylserine through the budding process to enhance infectivity. Virulence 16:2580150 41147668 10.1080/21505594.2025.2580150 PMC12604639

[35] Haiyilati A, Zhou L, Li J, Li W, Gao L, Cao H, Wang Y, Li X, Zheng SJ (2022) Gga-miR-30c-5p enhances apoptosis in Fowl Adenovirus Serotype 4-infected Leghorn Male Hepatocellular Cells and facilitates viral replication through Myeloid Cell Leukemia-1. Viruses 14:990 35632731 10.3390/v14050990 PMC9146396

[36] Zhang T, Yin C, Boyd DF, Quarato G, Ingram JP, Shubina M, Ragan KB, Ishizuka T, Crawford JC, Tummers B, Rodriguez DA, Xue J, Peri S, Kaiser WJ, Lopez CB, Xu Y, Upton JW, Thomas PG, Green DR, Balachandran S (2020) Influenza Virus Z-RNAs induce ZBP1-mediated necroptosis. Cell 180:1115–1129 32200799 10.1016/j.cell.2020.02.050 PMC7153753

[37] Christgen S, Zheng M, Kesavardhana S, Karki R, Malireddi RKS, Banoth B, Place DE, Briard B, Sharma BR, Tuladhar S, Samir P, Burton A, Kanneganti T (2020) Identification of the PANoptosome: a molecular platform triggering pyroptosis, apoptosis, and necroptosis (PANoptosis). Front Cell Infect Microbiol 10:237 32547960 10.3389/fcimb.2020.00237 PMC7274033

[38] Hartmann BM, Albrecht RA, Zaslavsky E, Nudelman G, Pincas H, Marjanovic N, Schotsaert M, Martinez-Romero C, Fenutria R, Ingram JP, Ramos I, Fernandez-Sesma A, Balachandran S, Garcia-Sastre A, Sealfon SC (2017) Pandemic H1N1 influenza A viruses suppress immunogenic RIPK3-driven dendritic cell death. Nat Commun 8:1931 29203926 10.1038/s41467-017-02035-9 PMC5715119

[39] Sorice M (2022) Crosstalk of autophagy and apoptosis. Cells 11:1479 35563785 10.3390/cells11091479 PMC9102887

[40] Hu Y, Wang B, Yi K, Lei Q, Wang G, Xu X (2021) IFI35 is involved in the regulation of the radiosensitivity of colorectal cancer cells. Cancer Cell Int 21:290 34082779 10.1186/s12935-021-01997-7 PMC8176734

[41] Shirai K, Shimada T, Yoshida H, Hayakari R, Matsumiya T, Tanji K, Murakami M, Tanaka H, Imaizumi T (2017) Interferon (IFN)-induced protein 35 (IFI35) negatively regulates IFN-beta-phosphorylated STAT1-RIG-I-CXCL10/CCL5 axis in U373MG astrocytoma cells treated with polyinosinic-polycytidylic acid. Brain Res 1658:60–67 28109979 10.1016/j.brainres.2017.01.018

[42] Tao Z, Na Y, Lulu M, Fengxiang X, Xiaobing L, Jiangwu H, Fei G, Ming L, Min F, Manman D (2025) Identification of Genes Stimulated by Type II Interferon in Ducks. Figshare. 10.6084/m9.figshare.30451406

