# Supplementary figures and images for: Identification of duck type II interferon-stimulated genes and revelation of duIFI35 inhibition of H5N6 AIV replication by promoting apoptosis
Source: Vet Res. 2026 Apr 16;57:89. doi: 10.1186/s13567-026-01747-5 (PMC13214279; doi:10.1186/s13567-026-01747-5)

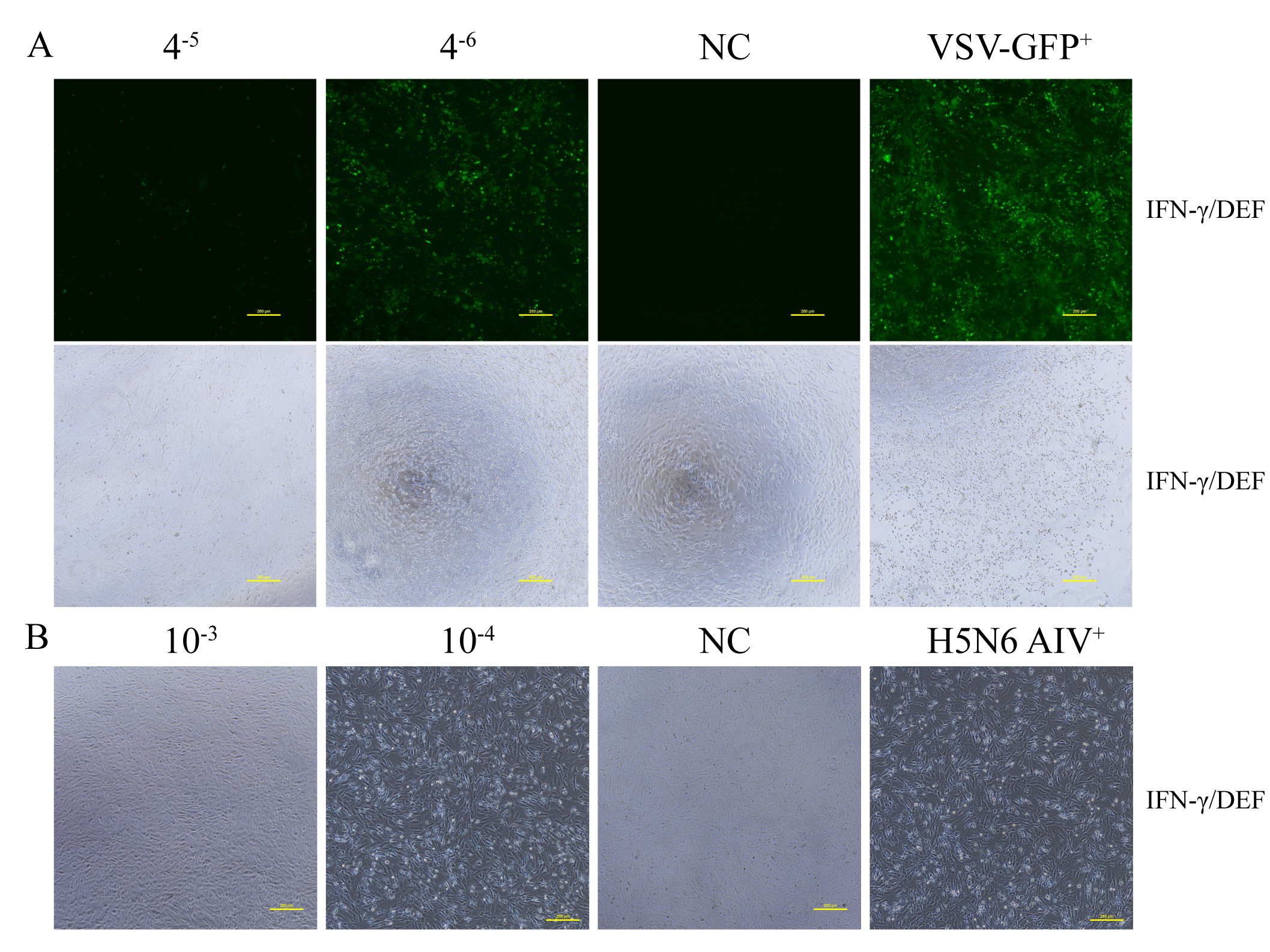

Supplement: Supplementary file 1 — Additional file 1 Activity detection of recombinant DuIFN-γ against vesicular stomatitis virus (VSV) and H5N6 AIV in vitro. (A) Cytopathic effects (CPE) caused by VSV (100 ¬TCID50) in DEFs preincubated with different concentrations of recombinant DuIFN-γ. Dilution factor (only the CPE results of two dilution factors at the critical point are shown): 4−5 (no CPE), 4−6 (CPE appearance). (B) CPE induced by H5N6 AIV in DEFs preincubated with different concentrations of recombinant DuIFN-γ. Dilution factor 10-3(no CPE), 10-4(CPE appearance). Abbreviations: NC, negative control (mock treated cells); VSV-GFP+, positive control (cells are directly inoculated with VSV without IFN treatment); H5N6 AIV+, positive control (cells are directly inoculated with H5N6 AIV without IFN treatment). [file 13567_2026_1747_MOESM1_ESM.tif]

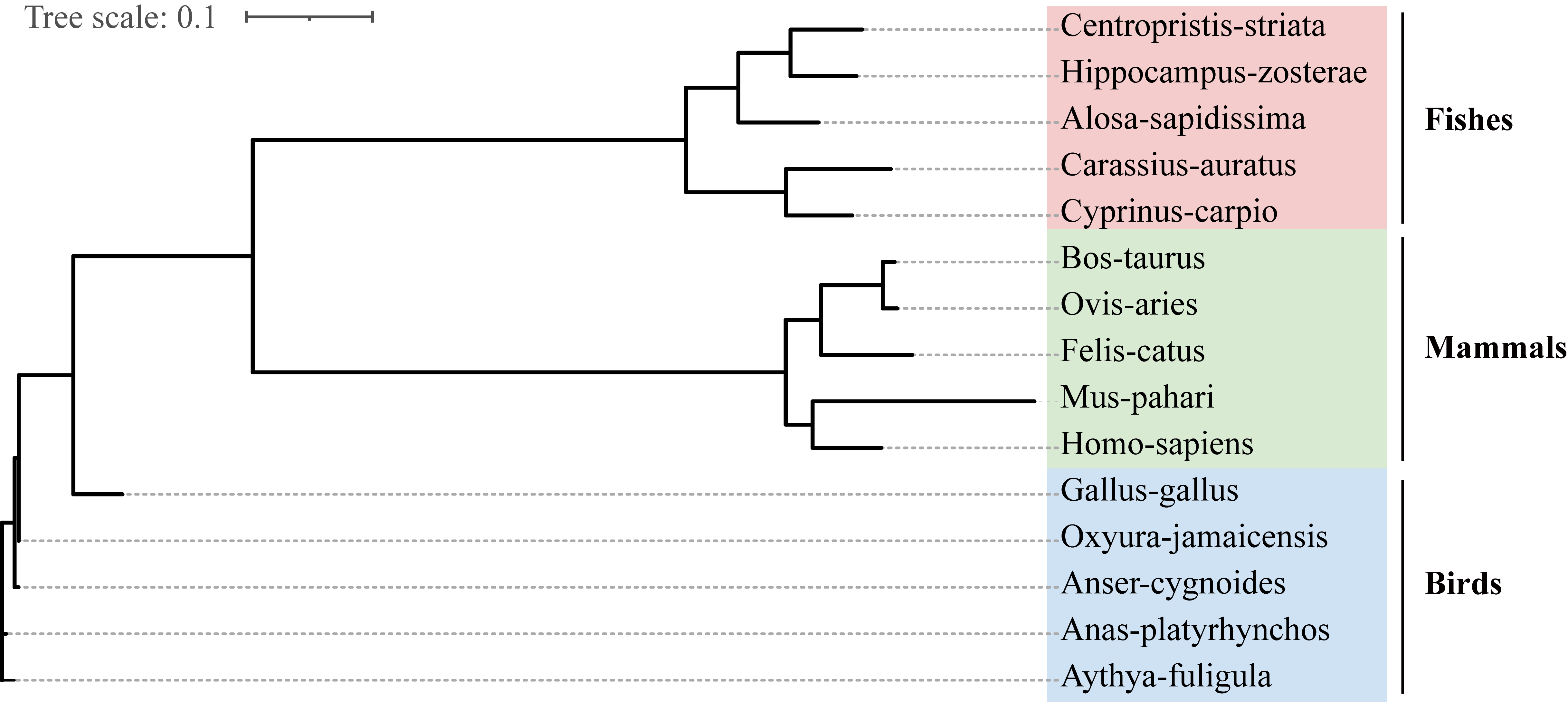

Supplement: Supplementary file 2 — Additional file 2 Phylogenetic tree constructed based on amino acid sequences of duCASP7 (XM_027459864.3) and CASP7 from other species. [file 13567_2026_1747_MOESM2_ESM.tif]

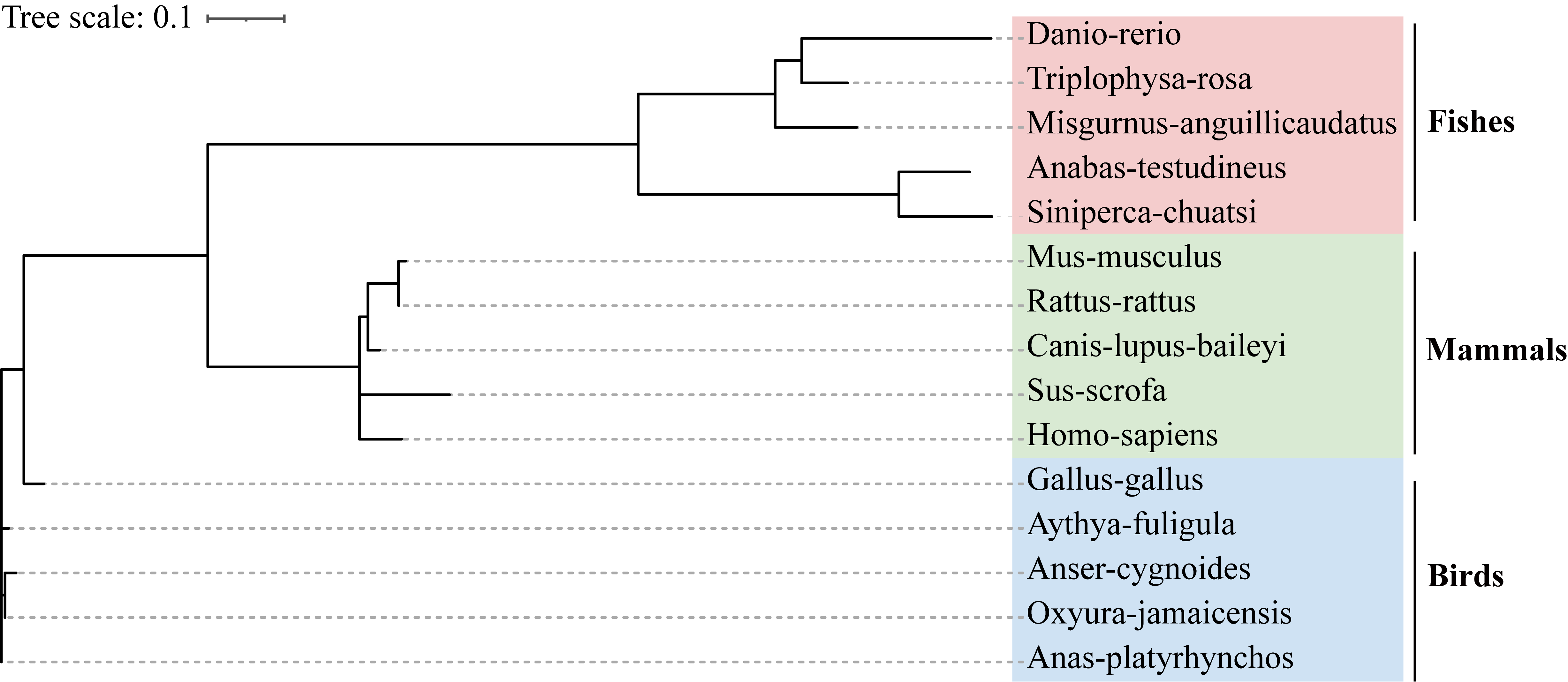

Supplement: Supplementary file 3 — Additional file 3 Phylogenetic tree constructed based on amino acid sequences of duGPX3 (XM_027468347.3) and GPX3 from other species. [file 13567_2026_1747_MOESM3_ESM.tif]

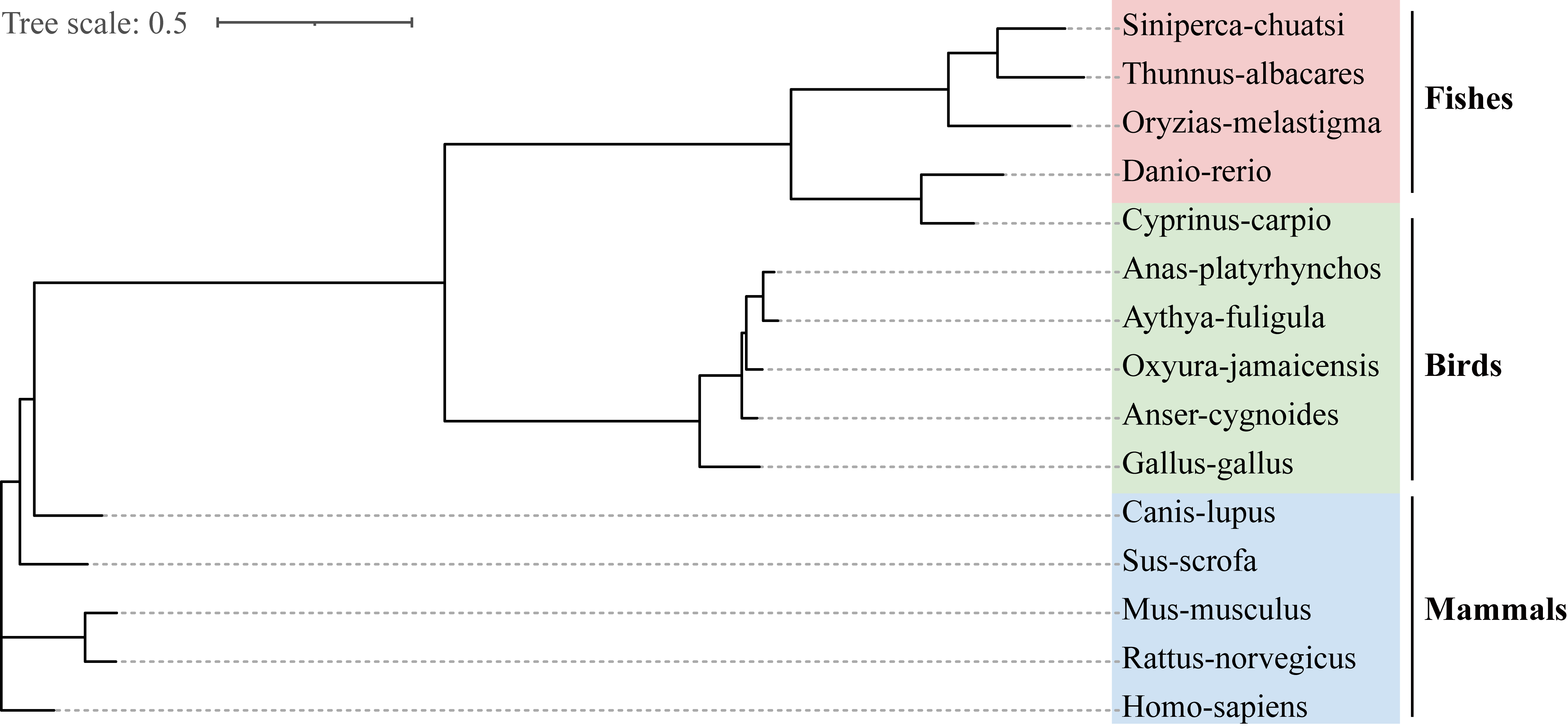

Supplement: Supplementary file 4 — Additional file 4 Phylogenetic tree constructed based on amino acid sequences of duIFI35 (XM_038168772.2) and IFI35 from other species. [file 13567_2026_1747_MOESM4_ESM.tif]

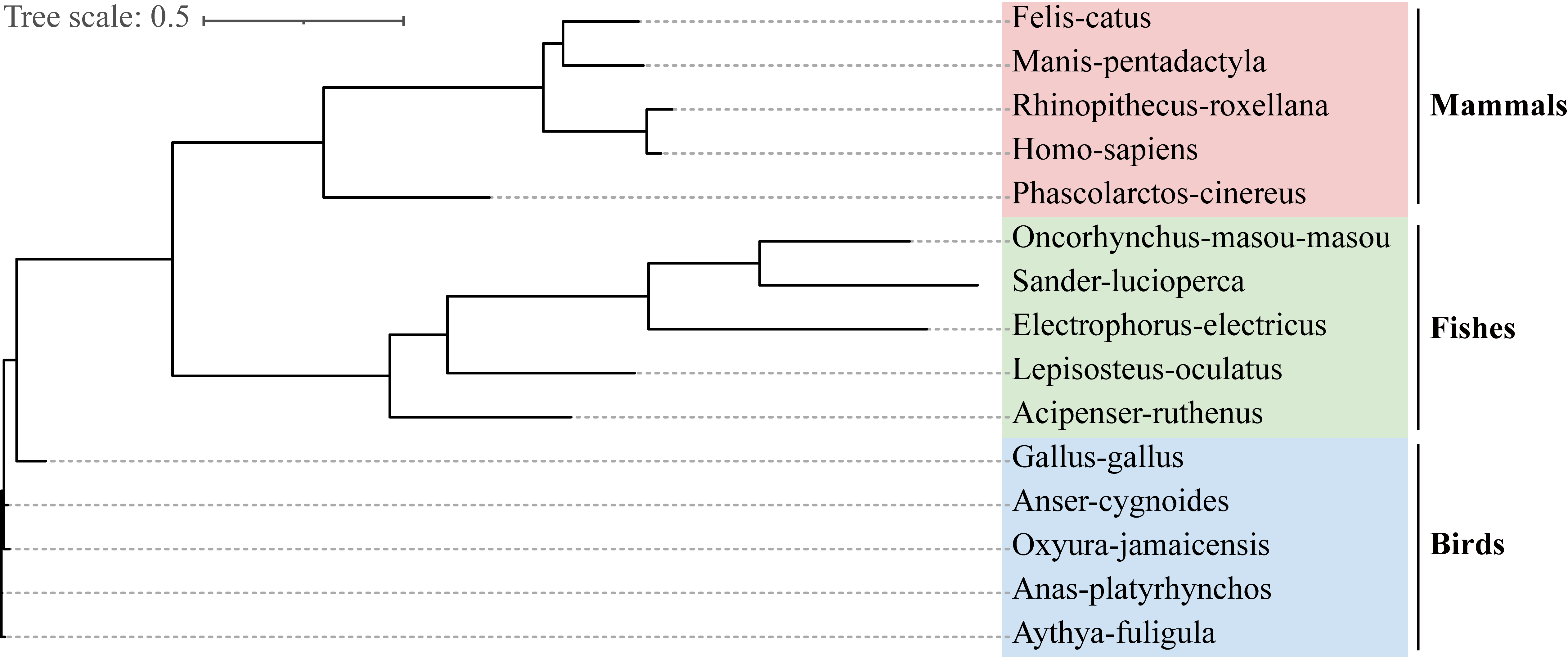

Supplement: Supplementary file 5 — Additional file 5 Phylogenetic tree constructed based on amino acid sequences of duIL17REL (XM_038173189.2) and IL17REL from other species. [file 13567_2026_1747_MOESM5_ESM.tif]

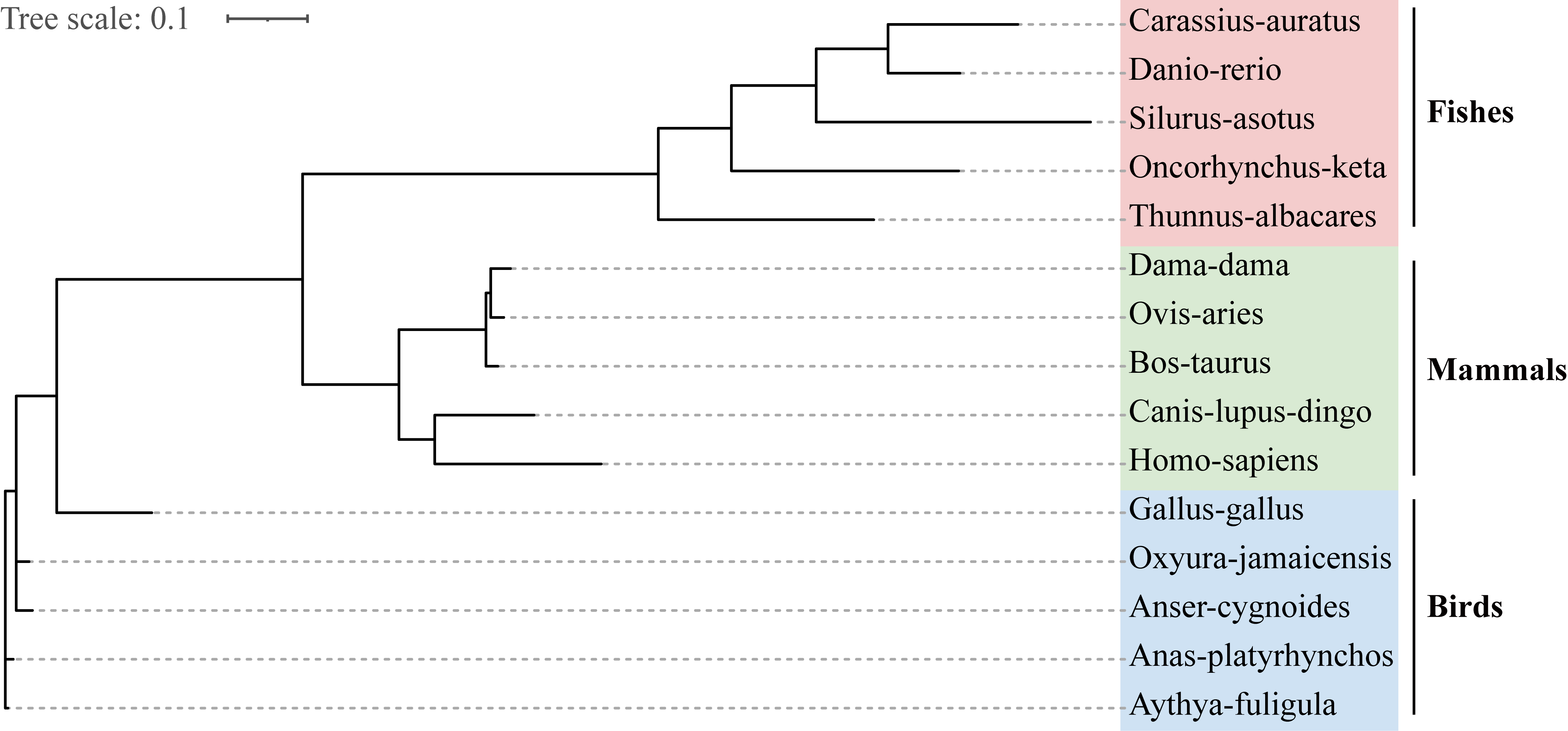

Supplement: Supplementary file 6 — Additional file 6 Phylogenetic tree constructed based on amino acid sequences of duRSAD2 (NM_001310801.1) and RSAD2 from other species. [file 13567_2026_1747_MOESM6_ESM.tif]

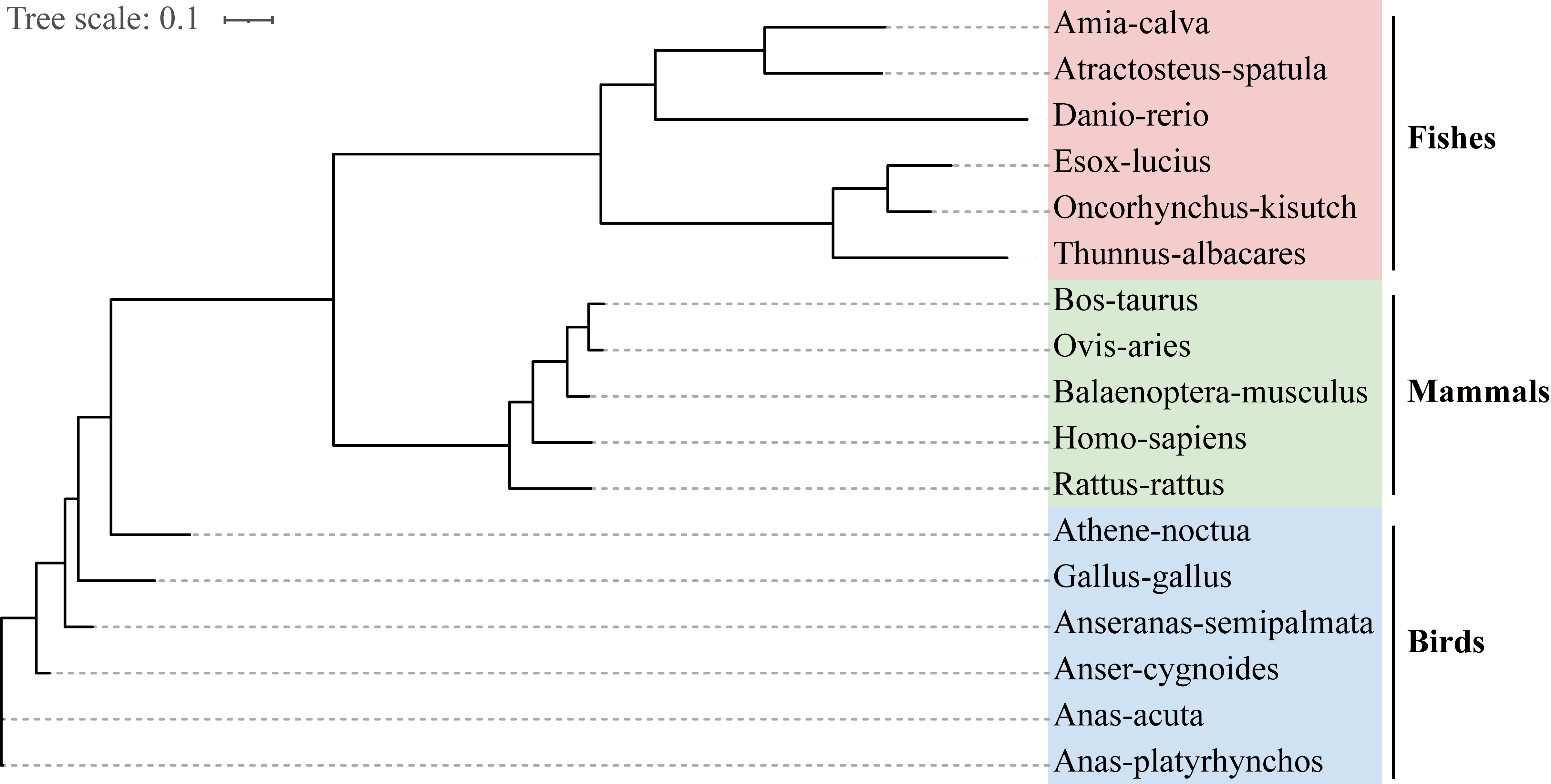

Supplement: Supplementary file 7 — Additional file 7 Phylogenetic tree constructed based on amino acid sequences of duSMAD9 (XM_005010927.6) and SMAD9 from other species. [file 13567_2026_1747_MOESM7_ESM.tif]

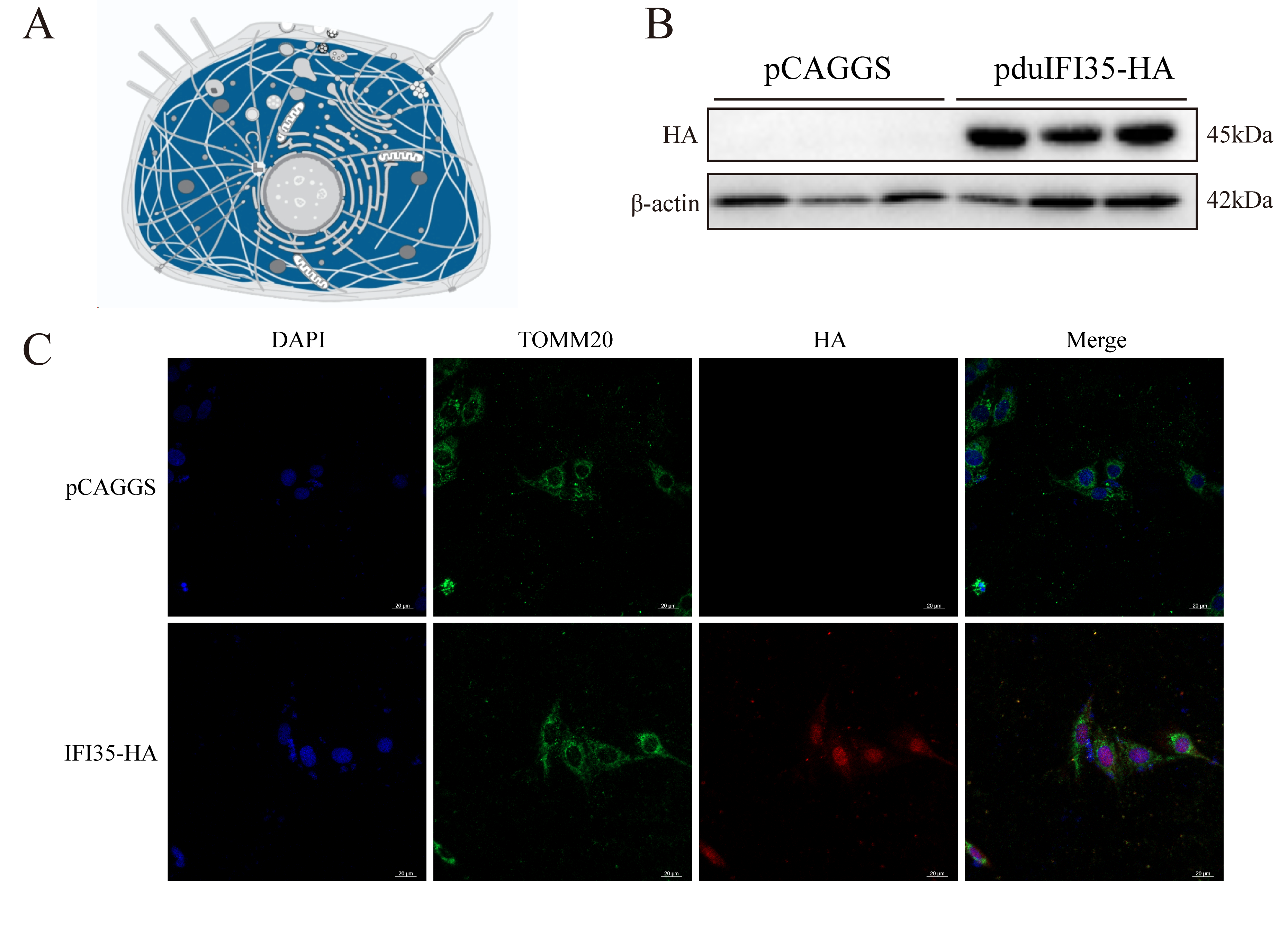

Supplement: Supplementary file 8 — Additional file 8 Expression of duIFI35 in DEFs. (A)WoLF PSORT II predicted the subcellular localization of duIFI35, showing its distribution (in blue) primarily in the cytoplasm. (B) Expression of pduIFI35-HA. (C) Subcellular localization of duIFI35 in DEFs. Red: anti-HA; Green: anti-TOMM20 (mitochondria); Blue: DAPI (nuclei). Scale bar, 20 μm. [file 13567_2026_1747_MOESM8_ESM.tif]

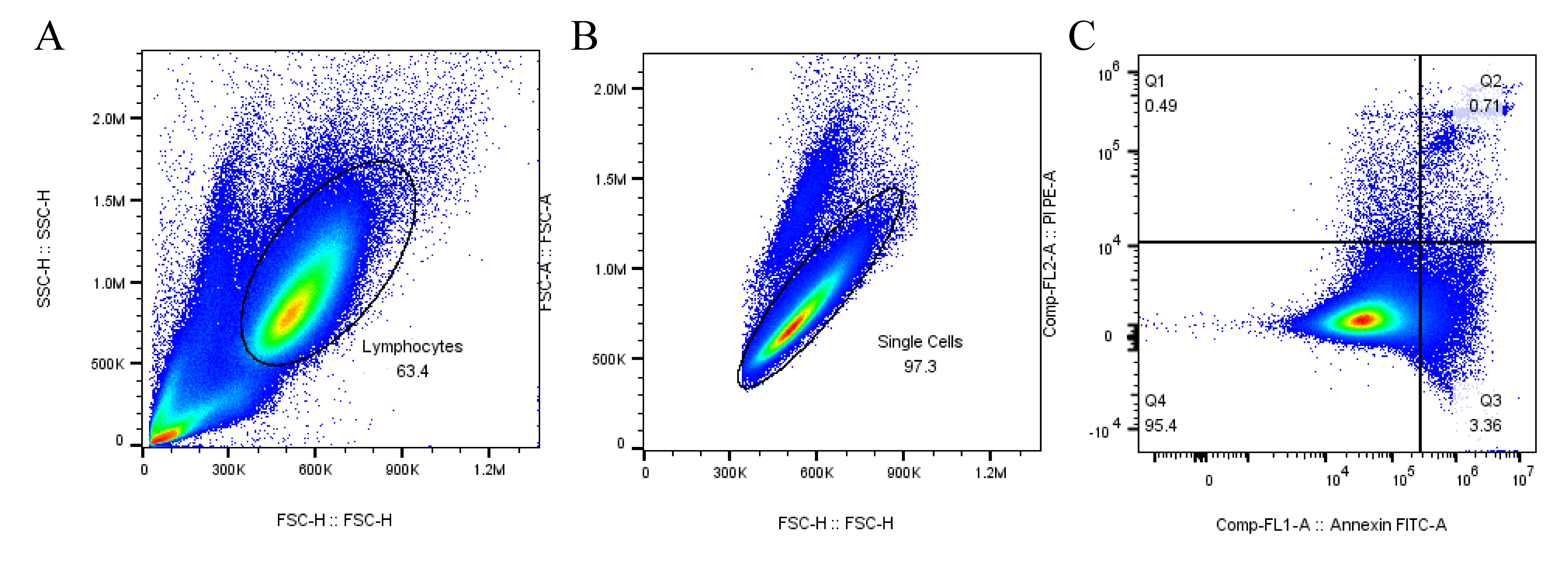

Supplement: Supplementary file 9 — Additional file 9 The gating strategy of apoptotic cells in DEFs. (A) Gating strategy of DEFs living cells. (B) Gating strategy of DEFs without adhesions. (C) Gating strategy of DEFs apoptotic cells. Q1, Q2 are dead cells, Q3 are apoptotic cells, and Q4 are living cells. [file 13567_2026_1747_MOESM9_ESM.tif]

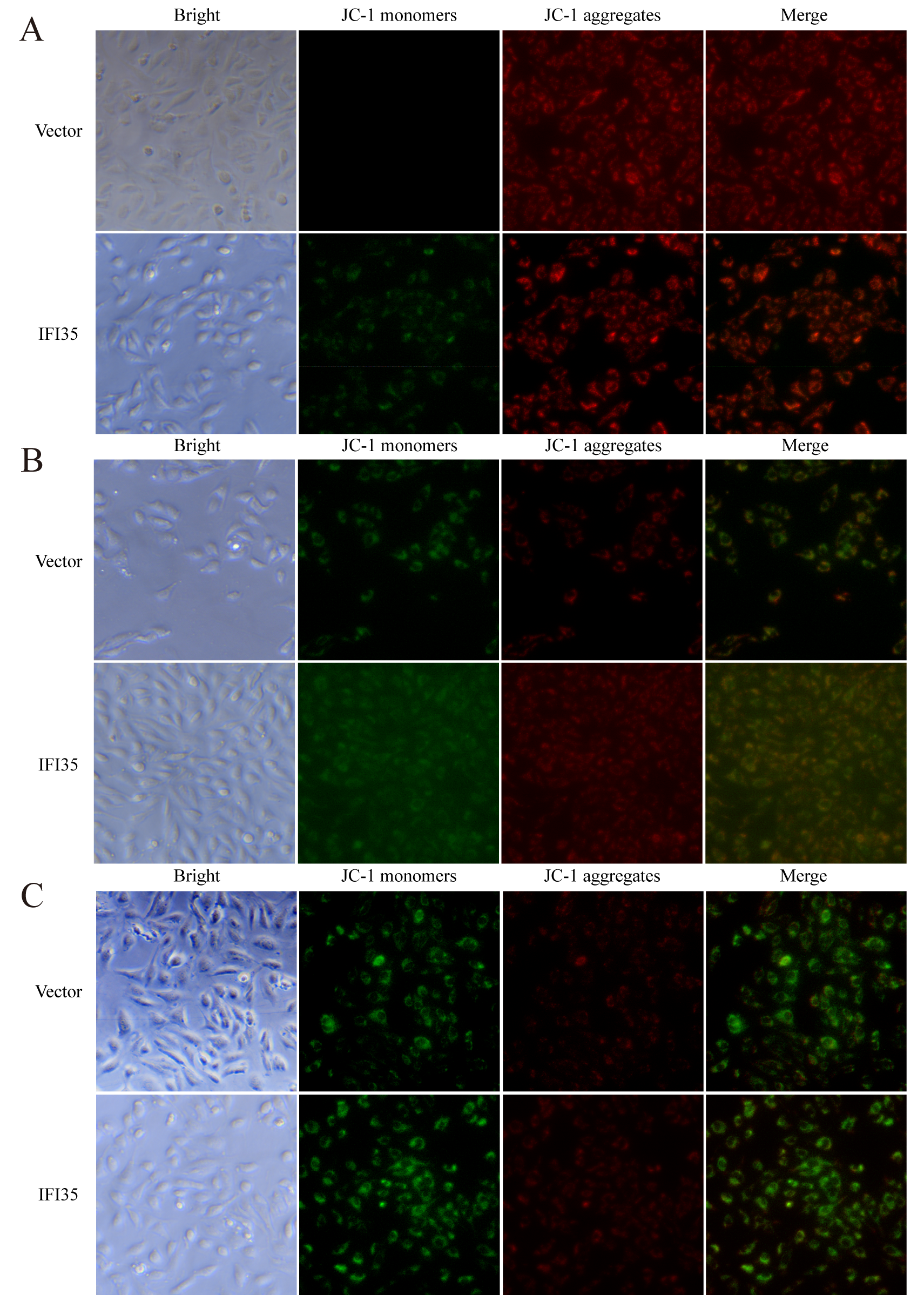

Supplement: Supplementary file 10 — Additional file 10 Overexpression of duIFI35 induces time-dependent mitochondrial depolarization in H5N6-infected DEFs. DEFs were transfected with empty vector or pCAGGS-duIFI35 for 24 h and subsequently infected with H5N6 virus. Mitochondrial membrane potential was assessed using JC-1 staining at (A) 12 h, (B) 24 h, and (C) 36 hpi. [file 13567_2026_1747_MOESM10_ESM.tif]
